# EDI3 knockdown in ER-HER2+ breast cancer cells reduces tumor burden and improves survival in two mouse models of experimental metastasis

**DOI:** 10.1186/s13058-024-01849-y

**Published:** 2024-05-30

**Authors:** Annika Glotzbach, Katharina Rohlf, Anastasia Gonscharow, Simon Lüke, Özlem Demirci, Brigitte Begher-Tibbe, Nina Overbeck, Jörg Reinders, Cristina Cadenas, Jan G. Hengstler, Karolina Edlund, Rosemarie Marchan

**Affiliations:** 1 Leibniz Research Centre for Working Environment and Human Factors at the TU Dortmund (IfADo), Ardeystrasse 67, 44139 Dortmund, Germany; 2 Department of Biology, Science Faculty, Dicle University, Diyarbakir, Turkey

**Keywords:** Breast cancer, HER2 positive breast cancer, Metastasis, Choline metabolism, Glycerophospholipid metabolism, GPCPD1, Anoikis

## Abstract

**Background:**

Despite progress understanding the mechanisms underlying tumor spread, metastasis remains a clinical challenge. We identified the choline-producing glycerophosphodiesterase, EDI3 and reported its association with metastasis-free survival in endometrial cancer. We also observed that silencing EDI3 slowed cell migration and other cancer-relevant phenotypes in vitro. Recent work demonstrated high EDI3 expression in ER-HER2+ breast cancer compared to the other molecular subtypes. Silencing EDI3 in ER-HER2+ cells significantly reduced cell survival in vitro and decreased tumor growth in vivo. However, a role for EDI3 in tumor metastasis in this breast cancer subtype was not explored. Therefore, in the present work we investigate whether silencing EDI3 in ER-HER2+ breast cancer cell lines alters phenotypes linked to metastasis in vitro, and metastasis formation in vivo using mouse models of experimental metastasis.

**Methods:**

To inducibly silence EDI3, luciferase-expressing HCC1954 cells were transduced with lentiviral particles containing shRNA oligos targeting EDI3 under the control of doxycycline. The effect on cell migration, adhesion, colony formation and anoikis was determined in vitro, and significant findings were confirmed in a second ER-HER2+ cell line, SUM190PT. Doxycycline-induced HCC1954-luc shEDI3 cells were injected into the tail vein or peritoneum of immunodeficient mice to generate lung and peritoneal metastases, respectively and monitored using non-invasive bioluminescence imaging. Metabolite levels in cells and tumor tissue were analyzed using targeted mass spectrometry and MALDI mass spectrometry imaging (MALDI-MSI), respectively.

**Results:**

Inducibly silencing EDI3 reduced cell adhesion and colony formation, as well as increased susceptibility to anoikis in HCC1954-luc cells, which was confirmed in SUM190PT cells. No influence on cell migration was observed. Reduced luminescence was seen in lungs and peritoneum of mice injected with cells expressing less EDI3 after tail vein and intraperitoneal injection, respectively, indicative of reduced metastasis. Importantly, mice injected with EDI3-silenced cells survived longer. Closer analysis of the peritoneal organs revealed that silencing EDI3 had no effect on metastatic organotropism but instead reduced metastatic burden. Finally, metabolic analyses revealed significant changes in choline and glycerophospholipid metabolites in cells and in pancreatic metastases in vivo.

**Conclusions:**

Reduced metastasis upon silencing supports EDI3’s potential as a treatment target in metastasizing ER-HER2+ breast cancer.

**Supplementary Information:**

The online version contains supplementary material available at 10.1186/s13058-024-01849-y.

## Background

Despite advances in early cancer detection and treatment, metastasis remains a major problem for tumor therapy and is responsible for approximately 90% of breast cancer-related deaths [1, 2]. The use of targeted therapies, such as trastuzumab or lapatinib in patients with human epidermal growth factor receptor 2 positive (HER2+) tumors that constitute approximately 20% of all breast cancers, has been tremendously successful [3, 4]. However, acquired and inherent resistance has limited the efficacy of HER2-targeting drugs [5], supporting the need for novel treatment options for this patient subset. In our most recent work, we demonstrated that expression and enzymatic activity of the glycerophosphodiesterase EDI3 is highest in HER2+ breast cancer cells that are also estrogen receptor negative (ER-), and in agreement, higher EDI3 mRNA expression was also observed in human ER-HER2+ breast cancers in an analysis of publicly available Affymetrix gene expression microarray datasets [6]. Knocking down EDI3 with siRNA in the ER-HER2+ cell lines, SKBR3 and HCC1954 revealed a significant loss in cell viability [6]. Furthermore, treating mice with the general phosphodiesterase inhibitor dipyridamole to pharmacologically inhibit EDI3 in vivo, resulted in a significant decrease in subcutaneous growth of tumors produced from HCC1954 cells. These findings were validated using an in vivo mouse model that allowed us to inducibly silence EDI3 in HCC1954 cells by the addition of doxycycline [6], which altogether support EDI3 as a potential treatment target in ER-HER2+ breast cancer.

EDI3 is encoded by the gene glycerophosphocholine phosphodiesterase 1 (*GPCPD1*) and is one of seven so far identified mammalian glycerophosphodiesterases (GDEs), each with its specific substrate and function in cells [7]. It is unique among the GDE family, because in addition to the enzymatic GDE domain—a common feature among all members—it is one of only three human proteins containing a carbohydrate binding moiety 20 (CBM20) domain. However, there is only little known about the physiological function of this ubiquitously expressed protein, with few existing studies demonstrating for example that EDI3 negatively regulates skeletal muscle differentiation in mice [8, 9] and has a role in muscle aging and age-associated glucose intolerance [10]. In our early work, we characterized EDI3’s enzymatic activity, demonstrating that it hydrolyzes glycerophosphocholine (GPC), a metabolic intermediate in phosphatidylcholine metabolism, to produce choline and glycerol-3-phosphate (G3P) [11], making it a central enzyme in choline metabolism, with links to phospholipid metabolism. More specifically, EDI3’s product, G3P is the backbone molecule of all glycerophospholipids, which are major structural components of cellular and vesicular membranes, critical for membrane stability, fluidity, and trafficking [12]. Downstream of EDI3, glycerol-3-phosphate acyltransferases esterify long-chain fatty acyl coenzyme A to G3P to produce the signaling lipid lysophosphatidic acid (LPA), and G3P may also be oxidized by glycerol-3-phosphate dehydrogenase to produce dihydroxyacetone phosphate (DHAP), which links EDI3 to glycolysis and gluconeogenesis. Thus, EDI3 acts at the intersection of several metabolic pathways critical for maintaining normal cell homeostasis, the deregulation of which may lead to pathologies including cancer.

Aberrant choline metabolism has indeed been reported in several malignancies [13–17] as extensively reviewed [18, 19]. More specifically elevated levels of choline-containing metabolites, including choline, phosphocholine (PCho) and GPC were shown in breast cancer cell lines [13, 20, 21] and tumor tissue [15, 22]. Higher PCho/GPC ratios have also been reported in more aggressive (e.g. MDA-MB-231) compared to less aggressive breast cancer cell lines (e.g. MCF7) [23], while a higher GPC/PCho ratio was seen in adjacent non-involved tissue compared to breast tumor tissues [24]. This deregulated cholinic phenotype promoted investigations into enzymes regulating choline metabolites as potential therapeutic targets, most prominently choline kinase that produces phosphocholine from choline and has been linked to tumor cell proliferation [25]. However, despite numerous reports of altered choline metabolism in cancer, only relatively little is published about the choline-producing enzyme EDI3 in this disease.

We were the first to describe a role for EDI3 in cancer, showing that high EDI3 expression is associated with metastasis and worse survival in human endometrial and ovarian cancer [11], and our earlier studies in multiple cell lines of different cancer types revealed that EDI3 is important in cell migration, adhesion, and spreading [11, 26, 27]. More recently, EDI3 was additionally reported by other groups to play a role in hypoxia-induced mitophagy in triple-negative breast cancer [28], and with sunitinib and paclitaxel resistance in renal and ovarian cancer, respectively [29, 30]. Our observation that EDI3 expression and enzymatic activity is highest in breast cancer cells of the ER-HER2+ subtype, as well as in human ER-HER2+ breast cancers [6], motivated us to focus on this specific molecular breast cancer subtype. We went on to demonstrate that EDI3 expression decreases upon silencing HER2 using siRNA or pharmacologically inhibiting HER2 with lapatinib or trastuzumab; increases after overexpressing NeuT, an oncogenic version of rat HER2; and is regulated downstream of PI3K, mTORC1 and GSK3β signaling pathways via transcription factors HIF1α, STAT3 and CREB [6]. In vitro, the loss of viability upon EDI3 silencing was found to be more pronounced in ER-HER2+ SKBR3 and HCC1954 cells compared to cells that are ER + HER2 + (BT474 and EFM192A) [6], suggesting that EDI3 may have specific roles in the different breast cancer subtypes. Importantly, the effect of silencing EDI3 on the viability of ER-HER2+ cells also differed from earlier findings in MCF7 (ER + HER2-) and MDA-MB-231 (ER-HER2-) cells, where loss of EDI3 had no effect on cell proliferation or viability [11].

While initial studies in mice showed that pharmacologically inhibiting or silencing EDI3 in vivo resulted in a significant decrease in the subcutaneous growth of tumors produced from ER-HER2+ HCC1954 cells [6], the effect of EDI3 on metastasis formation in vivo has not yet been studied. Therefore, in the present work, we investigated the impact of silencing EDI3 in ER-HER2+ breast cancer on features important for metastasis in vitro and metastatic burden in vivo using two mouse models of experimental metastasis.

## Materials and methods

### Cell culture

Human breast cancer cell line HCC1954 was purchased from the American Type Culture Collection (ATCC) and grown in RPMI1640 (stable Glutamine and 2.0 g/L NaHCO_3_, PAN, Biotech) supplemented with 10% fetal bovine serum (FBS, Thermo Fisher Scientific) and 1% sodium pyruvate (Sigma-Aldrich). Luciferase-expressing HCC1954 cells (HCC1954-luc) were additionally supplemented with 200 µg/ml Geneticin disulfate (G418) (Carl Roth). For the cultivation of HCC1954-luc shNEG and HCC1954-luc shEDI3, 10% tetracycline-free FBS (PAN Biotech) was used. Cell lines were authenticated by DSMZ according to the ANSI/ATCC ASN-0002–2011 guidelines and were regularly tested for mycoplasma using the Venor® GeM Classic kit (Minerva Biolabs).

### Transfection

Stable luciferase-expressing HCC1954 cells (HCC1954-luc) were generated by transfection with the firefly luciferase gene (luc2) encoding plasmid pGL4.51[luc2/CMV/Neo] (Promega) using the Lipofectamine 3000 Transfection Kit (Thermo Fisher Scientific). For the selection of transfected cells, 200 µg/ml geneticin disulfate (G418) (Carl Roth) was used.

### EDI3 silencing

To stably and inducibly silence EDI3, HCC1954-luc cells were transduced with SMARTvector™ lentiviral particles (Dharmacon) containing two shRNA oligos targeting different exons of EDI3 (Additional file 1: Suppl. Table S1A) under the control of a Tet-On 3G tetracycline-inducible system, as well as a non-targeting scrambled shRNA control, as previously described [6] and detailed in Suppl. Material and Methods (Additional file 2). For transient silencing, cells were transfected with siRNA oligos (Additional file 1: Suppl. Table S1A) and RNAiMax transfection reagent (Thermo Fisher Scientific) as described in Suppl. Material and Methods (Additional file 2).

### Gene and protein expression

Total RNA was isolated using the RNeasy Mini Kit (Qiagen), quantified with the NanoDrop N-2000 spectrophotometer (NanoDrop) and 2 µg of RNA was transcribed into cDNA using the High-Capacity cDNA Reverse Transcription Kit (Thermo Fisher Scientific). Expression was measured by qRT-PCR with QuantiFast SYBR Green (Qiagen), and QuantiTect primer assays (Additional file 1: Suppl. Table S1B) on the ABI 7500 Fast Real-Time PCR system (Applied Biosystems). Relative expression normalized to the house keeping gene ACTB was calculated using the 2^−ΔΔCT^ method. For protein, whole-cell lysates were collected in RIPA buffer containing protease and phosphatase inhibitors (Sigma Aldrich), and protein concentration was determined using the BCA Protein Assay Kit (Thermo Fisher Scientific). Protein samples were separated on SDS polyacrylamide gels using the Mini-PROTEAN Tetra Electrophoresis System (Bio-Rad) and transferred onto PVDF membranes (PerkinElmer). Protein expression was detected using antibodies listed in Suppl. Table S1C (Additional file 1).

### Cell migration

After 72 h treatment with 0.1 µg/ml doxycycline, 7 × 10^5^ HCC1954-luc shNEG and HCC1954-luc shEDI3 cells were re-plated into 2 well silicone inserts (Ibidi) and allowed to attach overnight. Culture inserts were removed using sterile tweezers, leaving a clean gap between the two cell monolayers. Directly upon removal of the insert, as well as on subsequent days, four photos per well were taken at the same position until the gap in the non-induced shNEG cells was almost fully closed. Alternatively, migration was investigated using scratch assays as described previously [11]. The size of the gaps was determined using the “wound healing tool” of the ImageJ software. The average percentage of “wound” closure was calculated from all four photographed positions and compared to the non-induced shNEG cells or cells transfected with scrambled siRNA (siNEG).

### Colony formation

Cells were treated with 0.1 µg/ml doxycycline either at the time of plating or 24 h post-plating. For this purpose, 500 untreated HCC1954-luc shNEG and HCC1954-luc shEDI3 cells were re-suspended in media containing doxycycline and plated into 6-well plates, or 500 untreated cells were plated, allowed to attach for 24 h and then treated with doxycycline. The media was either left unchanged for 14 days or replenished every three days with fresh media containing 0.1 µg/ml doxycycline. After 14 days, media was aspirated, cells were washed with 1 × PBS and subsequently fixed and stained for 20 min at RT in a 0.1% (w/v) crystal violet (Sigma Aldrich) solution containing 10% (v/v) ethanol. Cells from both treatment regimens were also collected and analyzed for EDI3 expression. Excess staining was removed by washing the wells thrice with tap water. After drying, pictures of each well were taken. The software ImageJ was used to determine the number and size of colonies.

### Adhesion

96-well plates were coated with 20 μg/ml human fibronectin (BD Biosciences) at 4 °C overnight. Excess fibronectin solution was removed, and the coated wells were allowed to dry before they were blocked with 1% bovine serum albumin (Carl Roth) in 1 × PBS at 4 °C overnight. Immediately before seeding, wells were washed once with 1 × PBS. After 72 h treatment with 0.1 µg/ml doxycycline, HCC1954-luc shNEG and HCC1954-luc shEDI3 cells were detached using 0.5% trypsin/EDTA (PAN-Biotech) and resuspended in serum-free media to which 0.5 mg/ml soybean trypsin inhibitor type II (Sigma) was added. Cells were pelleted by centrifugation for 5 min at 800×*g* at RT, resuspended in fresh serum-free media, and rotated for 1 h at 37 °C using the MacsMix rotator (Miltenyi Biotec). After rotation, cells were re-plated onto the fibronectin-coated 96-well plates (5 × 10^4^ cells per well) and incubated at 37 °C for different time periods. To visualize cell adhesion, the plate was washed once with 1 × PBS and adherent cells were stained and fixed with a 0.1% (w/v) crystal violet (Sigma Aldrich) solution containing 10% (v/v) ethanol for 20 min at RT. Excess staining solution was removed by rinsing the plate carefully with tap water and drying overnight at RT. Photos of the stained cells were taken with a phase-contrast microscope using a 10× objective. For quantification, cells were destained with 0.2% Triton X-100 for 20 min on an orbital shaker at RT and absorption was measured at 570 nm in a plate reader (Infinite M200 Pro, Tecan).

### Anoikis

Cell culture plates were coated with poly(2-hydroxyethyl methacrylate) (poly-HEMA), a polymer that prevents cell adhesion. Briefly, poly-HEMA (Sigma Aldrich) solution of 10 mg/ml was prepared in 95% ethanol by shaking vigorously at 37 °C overnight. After sterile filtration using a 0.2 µm filter, 6-well plates were coated with 750 µl poly-HEMA solution per well and left to dry overnight. Before plating cells, plates were washed with 1 × PBS. HCC1954-luc shNEG and HCC1954-luc shEDI3 cells were incubated with 0.1 µg/ml doxycycline and SUM190PT cells were transfected with siRNA oligos 72 h before cells were replated into poly-HEMA coated 6-wells (5 × 10^5^ cells per well). After 24 or 48 h, 100 µl CellTiter-Blue® reagent of the Cell Viability Assay (Promega) was added per well to determine cell viability according to the manufacturer’s instructions, and fluorescence was measured at 579Ex/584Em nm in a plate reader (Infinite M200 Pro, Tecan).

### In vivo experiments

HCC1954-luc shNEG and HCC1954-luc shEDI3 cells were induced with 0.1 µg/ml doxycycline 72 h prior to injection. 1 × 10^6^ cells with and without doxycycline treatment (in 100 µl 1 × PBS) were injected either into the tail vein or into the peritoneum of five to seven, six- to eight-week-old female CD1 nude mice (Charles River) per condition. Mice injected with doxycycline-induced cells were fed a diet containing 625 mg/kg doxycycline (Ssniff) ad libitum. Non-invasive bioluminescence imaging was performed weekly. For this purpose, mice were injected intraperitoneally with 150 mg/kg body weight D-luciferin (PerkinElmer) and anesthetized by inhalation of isoflurane in oxygen (2.5% (v/v)) at a flow of 1 l/min. Ten minutes after luciferin injection, the luminescent signal was measured by a cooled charge-coupled device (CCD) camera using the IVIS Spectrum In Vivo Imaging System (PerkinElmer). For quantitative analysis, the total signal (photons/sec) in the regions of interest was determined using Living Image® 4.7.2 software (PerkinElmer). Mice were observed daily, and their overall condition was scored and tallied according to the conditions stipulated in the scoresheet, such as weight loss, ascites formation, abnormal behavior, and unusual physical appearance. Once terminal endpoints were reached, mice were sacrificed.

For the timed organ collection six and eight weeks after intraperitoneal injection of the cells, mice were sacrificed by cervical dislocation. Ascites fluid, if present, was collected with a syringe and liver, diaphragm, kidneys, the complex of stomach, spleen, and pancreas, as well as the two gonadal white adipose tissues were excised and washed in 1 × PBS to remove residual ascites fluid. The organs were positioned on a black non-reflecting Lexan foil next to a drop of 100 µl of the collected ascites fluid. Luminescence signals were measured in the IVIS Spectrum imager approx. 20 min after D-luciferin injection to localize tumors in the organs and to quantify viable cells within the ascites fluid. All animal studies were performed in accordance with the guidelines stipulated by the Society of Laboratory Animal Science (81–02.04.2020.A261, Gesellschaft für Versuchstierkunde, GV-SOLAS).

### Metabolite analysis

To extract metabolites, cell culture dishes with six technical replicates (wells from a six-well plate) were placed on ice, medium was aspirated, cells were washed with ice cold 1 × PBS thrice, and snap-frozen with liquid nitrogen. Ice cold methanol spiked with internal standards was added to the wells. Cells were scraped, collected, and kept on ice. All extracts were stored at −80 °C until further processing. Replicate wells for all conditions were used to determine the cell number per well with the CASY-TT cell counter. The extracted metabolite samples were fractionated using the simultaneous metabolite, protein, lipid extraction (SIMPLEX) protocol [31] (Additional file 2: Suppl. Material & Methods). Lipids and metabolites were subsequently measured by targeted LC–MS/MS. Details of the LC–MS/MS analyses are given in Suppl. Material and Methods (Additional file 2) and Suppl. Table S2 (Additional file 3). All LC–MS/MS data were interpreted using the Skyline Daily software [32].

### Matrix-assisted laser desorption ionization (MALDI) mass spectrometry imaging (MSI)

Macroscopically visible nodules were excised, when technically possible, from the complex of stomach, spleen, and pancreas, weighed, rapidly frozen in liquid nitrogen and stored at -80 °C. The frozen specimens were sectioned serially into 5 µm slides at -18 °C using a NX70 cryostat (Thermo Fisher Scientific), thaw-mounted on IntelliSlides (Bruker Daltonics) and dried in a desiccator.

For MALDI-MSI, the slides were first sprayed (four layers) with 10 mg/mL α-cyano-4-hydroxy-cinnamic acid (Sigma Aldrich) in 50% acetonitrile using an HTX Imaging-Sprayer (HTX Technologies LLC) at 30 °C at 10 psi nitrogen and a flow rate of 150 µL/min. MALDI-measurements were then conducted with 20 µm spatial resolution (400 shots per pixel) in positive mode with a timsTOFfleX (Bruker Daltonics) without ion-mobility separation in a mass range from 60–1,200 m/z and calibrated to red phosphorous. Data were interpreted using Scils Lab MVS, Version 2024 a Pro. The slides were subsequently stained with HER2 antibody to identify tumor-positive regions (described below). The mass of 706.54 + (candidate molecule PC 30:0) correlated in a large part with HER2 staining (Additional file 4: Suppl. Figure S1) and was used to generate tumor-cell-containing areas-of-interest, which were in turn used for the differential analysis of the [M + H] + signals (± 10 ppm) of the choline-related metabolites (GPC, phosphocholine and choline). The identity of these metabolite signals were verified by MS/MS-analyses. Measurements with low total ion current (TIC) (< 5% of mean), low tumor cell content (< 20% HER2 positive staining,) or blood contamination were excluded from the analysis, leading to six MALDI-MS-images from each group passing internal quality control that were then used for further analysis. Peak areas of the signals of interest were extracted and TIC-normalized [33]. Statistical comparison was done by two-tailed Student’s t-test.

### Immunohistochemistry

Cryosections scanned by MALDI-MSI were subsequently analyzed by automated IHC using the Discovery Ultra Automated Slide Preparation System (Roche). Slides were incubated in 4% formaldehyde for 4 min followed by pretreatment in demasking buffer (pH 8.46) at 95 °C for 4 min. Endogenous peroxidase activity was blocked by incubation with inhibitor CM (Roche) for 8 min. The HER2 rabbit monoclonal antibody 134,182 (Abcam) was applied at 1:2000 dilution for 60 min at 37 °C. For visualization of antibody binding the slides were subsequently incubated with UMap anti-rabbit HRP (Roche) for 16 min followed by treatment with DAB staining solution for 8 min. For counter-staining of the nuclei, Hematoxylin II and Bluing Reagent were used for 8 min and 4 min, respectively. Subsequently, the sections were dehydrated in a graded ethanol series and then incubated twice in rotihistol for 3 min each. Finally, the slides were mounted with Entellan® (Sigma Aldrich) and scanned using an AxioScan Z.1.

### Statistics

Analyses were performed using GraphPad Prism, version 9.4. Unless stated in the respective figure legends, data are presented as mean ± SD. Two-sided Student’s t-test was used to determine statistical significance, and P < 0.05 was considered significant.

## Results

### Increased GPC/PCho ratio after EDI3 knockdown

**Fig. 1:**
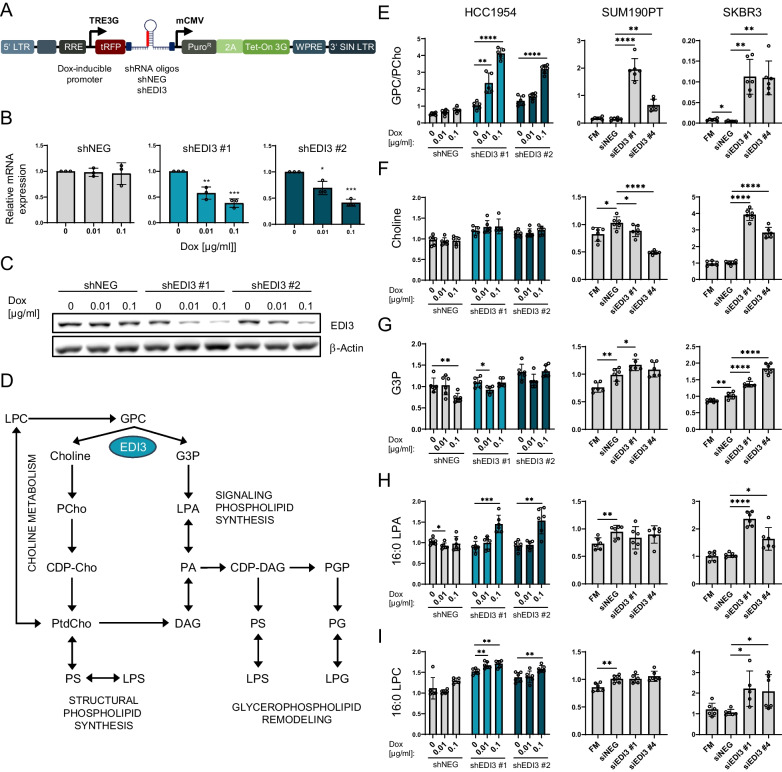
EDI3 silencing alters glycerophospholipid metabolism in ER-HER2+ breast cancer cells. **A** Schematic of the inducible lentiviral shRNA vector (SMARTvector™, Dharmacon) for doxycycline-dependent expression of shRNA oligos. **B** EDI3 mRNA expression and **C** representative Western blot showing EDI3 protein expression in HCC1954-luc shNEG and HCC1954-luc shEDI3 (oligos shEDI3 #1 and #2) cells after 72 h treatment with 0.01 or 0.1 µg/ml doxycycline. **D** Overview of the metabolic pathways downstream of EDI3. By hydrolyzing GPC to choline and G3P, EDI3 plays a role in choline metabolism via the Kennedy pathway as well as in the synthesis of signaling and structural glycerophospholipids. **E** Intracellular GPC/PCho ratio, and intracellular levels of **F** choline, **G** G3P, **H** 16:0 LPA and **I** 16:0 LPC measured using LC–MS/MS in HCC1954-luc shNEG and shEDI3 after 72 h treatment with 0.01 or 0.1 µg/ml doxycycline (left panel) as well as in SUM190PT (middle panel) and SKBR3 cells (right panel) after silencing EDI3 using two different siRNA oligos. Metabolite levels were determined by calculating the ratios of the integrated peaks of the endogenous metabolites and the internal standards. Quantities of metabolites were normalized to cell number and presented relative to untreated control cells (shNEG or siNEG). Data represent mean ± SD from at least five technical replicates (wells from a six-well plate) from one or two independent experiments (*p < 0.05; **p < 0.01; ***p < 0.001; ****p < 0.0001). GPC, glycerophosphocholine; PCho, phosphocholine; LPA, lysophosphatidic acid; PA, phosphatidic acid; LPC, lysophosphatidylcholine; LPS, lysophosphatidylserine; LPG, lysophosphatidylglycerol; DAG, diacylglycerol; PtdCho, phosphatidylcholine; CDP-Cho, cytidine diphosphate choline; PS, phosphatidylserine; G3P, glycerol-3-phosphate; CDP-DAG, cytidine diphosphate diacylglycerol; PGP, phosphatidylglycerol phosphate; PG, phosphatidylglycerol

To study the impact of EDI3 on processes that may be important for tumor metastasis in ER-HER2+ breast cancer, we used luciferase-positive HCC1954 cells into which we stably transfected doxycycline-inducible shRNA constructs (Fig. 1A) [6]. First, doxycycline-dependent downregulation of EDI3 expression was confirmed on both RNA (Fig. 1B) and protein (Fig. 1C) levels. We then investigated the effect of EDI3 silencing on intracellular metabolites linked to EDI3 (Fig. 1D). Knocking down EDI3 resulted in a strong dose-dependent increase in the levels of its substrate, GPC (Additional file 5: Suppl. Figure S2A), indicating reduced enzymatic activity as previously described in other cell lines [11, 27], and a significantly increased GPC/PCho ratio (Fig. 1E), reversing the low GPC/PCho ratio reported in more aggressive cell lines [13, 17]. Choline and G3P levels were not significantly altered downstream of EDI3 (Fig. 1F, G), nor was there a consistent change in PCho or betaine (Additional file 5: Suppl. Figure S2A), which may be due to compensation by alternative choline sources, or uptake from the extracellular media by choline transporters. To study the effect of EDI3 knockdown in additional ER-HER2+ cell lines, we used siRNA to silence EDI3 in SUM190PT and SKBR3 cells (Additional file 6: Suppl. Figure S3A-B). Importantly, we could confirm an increase in the intracellular GPC/PCho ratio as well as GPC levels (Fig. 1E, Additional file 5: Suppl. Figure S2B-C) matching the findings described for HCC1954. The change in levels of EDI3’s direct downstream products, choline and G3P (Fig. 1F, G) were not consistent among the three ER-HER2+ cell lines; nor did we observe consistent changes in the levels of PCho or betaine, which are both produced using choline as a substrate (Additional file 5: Suppl. Figure S2B-C).

Via its downstream product G3P, EDI3 is also linked to the synthesis of structural and signaling glycerophospholipids (Fig. 1D) that are needed to maintain homeostasis and deregulated in cancer. In our previous work, we showed that silencing EDI3 in the ER + HER2- MCF7 cell line reduced the levels of signaling lipids lysophosphatidic acid (LPA) and phosphatidic acid (PA) [11, 27]. In addition, we also demonstrated a relationship between intracellular LPA levels and migration in MCF7 cells [27].

Therefore, in the present study, the levels of 71 glycerophospholipids were measured using targeted LC–MS/MS after silencing EDI3 in the three ER-HER2+ cell lines (Fig. 1H-I; Additional file 7: Suppl. Figure S4). In contrast to previous findings in MCF7 cells, knocking down EDI3 in HCC1954-luc and SKBR3 cells significantly increased intracellular LPA and PA levels (Fig. 1H; Additional file 7: Suppl. Figure S4), which was mostly consistent for the three LPA and five PA species detected. In both cell lines, there were also increased levels of almost all lysophosphatidylcholine (LPC) species measured, which can be deacylated to GPC or acylated to PtdCho (Fig. 1I; Additional file 7: Suppl. Figure S4); levels of the latter were also higher compared to shNEG/siNEG controls. Other glycerophospholipids were also investigated (Additional file 7: Suppl. Figure S4), namely phosphatidylglycerol (PG), phosphatidylserine (PS), phosphatidylethanolamine (PE) and phosphatidylinositol (PI), as well as the lysophospholipids, lysophosphatidylglycerol (LPG), lysophosphatidylserine (LPS), lysophosphatidylethanolamine (LPE) and lysophosphatidylinositol (LPI), all of which are involved in remodeling of phospholipid membranes [12]. Silencing EDI3 in HCC1954-luc and SKBR3 increased the levels of many of the above-listed glycerophospholipids (Additional file 7: Suppl. Figure S4). This increase was consistent between both cell lines for most species of PG, LPG, PE, and PI. However, for the other measured lipid species, the increase was variable depending on cell lines, lipid species, siRNA or shRNA oligo or doxycycline concentration (Additional file 7: Suppl. Figure S4). Conversely, in SUM190PT cells most of the analyzed glycerophospholipids either decreased—such as reduced levels of almost all species of LPA, PA, and PtdCho, or did not change after EDI3 silencing (Fig. 1H, I; Additional file 7: Suppl. Figure S4).

All the above-mentioned phospholipids are linked to cellular processes relevant in the process of metastasis, including cell migration [34–42], proliferation [34, 36, 39, 41] and adhesion [36, 42]. Therefore, the strong increase in the GPC/PCho ratio upon EDI3 silencing as well as the observed alterations in levels of signaling phospholipids prompted us to further investigate EDI3’s role in metastasis-relevant processes in vitro.

### EDI3 knockdown in HCC1954 cells does not affect migration but reduces colony formation

**Fig. 2:**
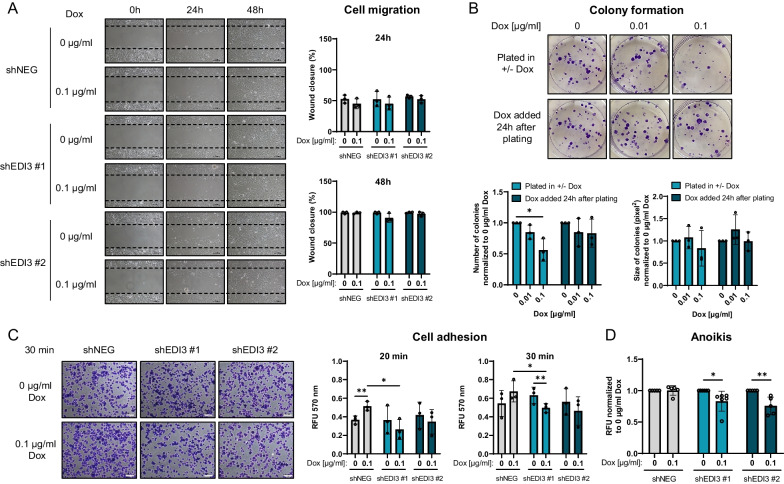
Doxycycline-induced EDI3 silencing influences colony formation, cell adhesion and anoikis resistance. **A** Representative images (left panel) and corresponding quantification (right panel) of the wound healing assay with HCC1954-luc shNEG and shEDI3 cells treated with 0.1 µg/ml doxycycline. **B** Representative images (upper panel) and corresponding quantification (lower panel) of colonies (number and size) formed by HCC1954-luc shEDI3 cells treated with 0.01 or 0.1 µg/ml doxycycline at the time of (plated in ± Dox) or after (Dox added 24 h after plating) plating. **C** Representative pictures (left) of HCC1954-luc shNEG and shEDI3 cells stained with crystal violet after 30 min of adhesion to a fibronectin matrix and quantification (right panel) shown as RFU after 20 and 30 min measured at 570 nm after destaining. **D** Viability (RFU) of HCC1954-luc shNEG and shEDI3 cells induced with 0.1 µg/ml doxycycline relative to non-induced control cells (0 μg/ml doxycycline) measured 24 h after plating on a poly-HEMA matrix as a measure of anoikis resistance. Scale bars represent 100 μm. Values in graphs represent mean ± SD from at least three independent experiments (*p < 0.05; **p < 0.01). RFU, relative fluorescence units

Our earlier work demonstrated a role for EDI3 in migration in several different cell lines [11, 27], although none were HER2+ breast cancer cells. Therefore, in the present work, we first used our doxycycline-inducible model of ER-HER2+ breast cancer to study EDI3’s influence on cell migration using the in vitro wound healing assay. Interestingly, no difference in wound closure was observed between induced and non-induced HCC1954-luc shEDI3 cells (Fig. 2A). Consistently, silencing EDI3 in HCC1954 cells using siRNA targeting different exons of EDI3 also had no significant effect on migration (Additional file 8: Suppl. Figure S5). Therefore, we focused on other endpoints known to be relevant in the metastasis process.

The colony formation assay can recapitulate different steps in the metastasis process, including initial attachment, flattening, or spreading, and proliferation of single cells to form colonies. We recently reported that doxycycline-inducible EDI3 silencing in HCC1954 cells led to a significant reduction in the number of colonies when silencing was induced 72 h prior to plating the cells [6]. These findings indicate that EDI3 may be involved in any one of the steps involved in colony formation. Thus, to examine in which step EDI3 is most relevant, we treated HCC1954-luc shEDI3 cells with doxycycline either at the time of plating, or 24 h after plating. The former approach aims to investigate EDI3’s influence on initial attachment and proliferation of the cells, while the latter focuses on EDI3’s role in the proliferation of single cells since EDI3 is silenced after the cells are already attached. Knockdown was confirmed after 14 days (Additional file 9: Suppl. Figure S6A) and quantitative analysis revealed that inducing EDI3 knockdown during the plating step resulted in a significant reduction in colony number for at least one concentration of doxycycline, while silencing EDI3 24 h after re-plating had no effect (Fig. 2B). Replenishing the media with fresh doxycycline every three days did not additionally alter EDI3 expression nor the number or size of colonies compared to the condition where media was unchanged over the assay period (Additional file 9: Suppl. Figure S6A-B). Since the most prominent effect on colony formation was observed when EDI3 was silenced 72 h prior to plating the cells [6], and only a minor or no effect was seen when EDI3 was silenced during or after cell plating, the results altogether suggest that EDI3 is important during the early phase of colony formation.

### Silencing EDI3 reduces cell adhesion and increases susceptibility to anoikis

To determine if the reduction in colony formation upon silencing EDI3 is caused by impaired adhesion, EDI3’s influence on attachment was analyzed by testing the ability of cells to attach onto a fibronectin matrix. Silencing EDI3 with 0.1 µg/ml doxycycline in the HCC1954-luc shEDI3 cells led to reduced cell adhesion, which was significant with one shRNA oligo after 20 and 30 min compared to doxycycline-treated shNEG cells (Fig. 2C). Notably, doxycycline treatment significantly increased attachment of HCC1954-luc shNEG cells 20 min after seeding (Fig. 2C). While these results suggest that silencing EDI3 reduces adhesion, the true effect may be stronger than what is observed because it may be obscured by the adhesion-promoting effect of doxycycline in this model.

In addition to adhesion, we also examined if EDI3 influences processes prior to cell attachment, namely the ability of cells to survive in suspension and escape anoikis. Here, doxycycline-induced HCC1954-luc shNEG and shEDI3 cells were plated onto a poly-HEMA matrix which prevents attachment. Silencing EDI3 with two different shRNA oligos significantly reduced viability while doxycycline treatment alone had no effect on the viability HCC1954-luc shNEG cells (Fig. 2D), indicating that a reduction in EDI3 makes the cells more susceptible to anoikis. Increased susceptibility to anoikis upon EDI3 silencing was confirmed in a second ER-HER2+ cell line, SUM190PT using three different siRNA oligos (Additional file 10: Suppl. Figure S7A-C). We also observed reduced viability in adherent SUM190PT cells upon silencing EDI3 (Additional file 10: Suppl. Figure S7D), similar to our previous findings in HCC1954 cells [6]. As anoikis is an important factor for cells that are in circulation during the process of metastasis, as is adhesion for the colonization process, these findings encouraged us to investigate EDI3 in a mouse model of metastasis.

### Reduced luminescence upon EDI3 silencing in two mouse models of experimental metastasis

**Fig. 3:**
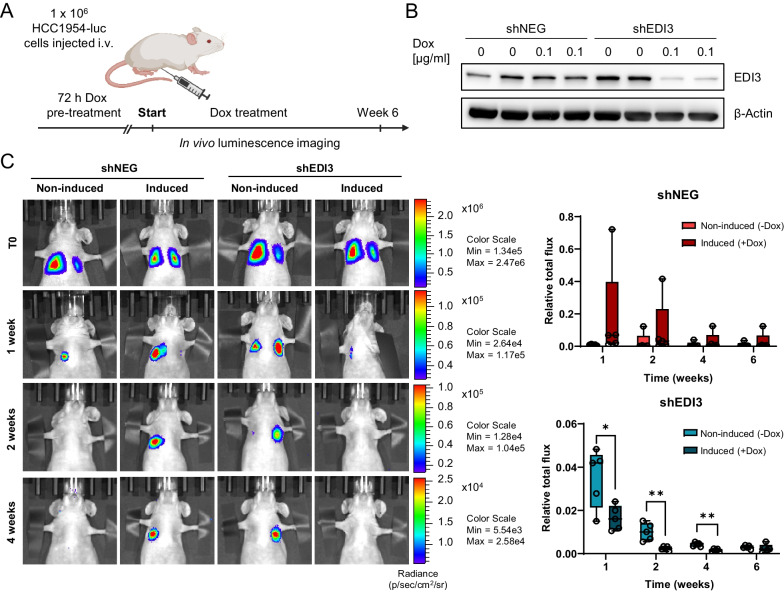
Doxycycline-induced EDI3 knockdown in HCC1954 results in reduced formation of lung metastasis in mice. **A** Schematic illustration of the experimental plan. Luciferase-expressing HCC1954 shNEG and shEDI3 cells were induced with doxycycline for 72 h, followed by tail vein injection into doxycycline pre-treated and untreated CD1 nude mice, respectively. Doxycycline was administered to the mice by a 625 mg/kg doxycycline containing diet (Ssniff) ad libitum. Luminescence signal was measured over six weeks. **B** Western blot confirming EDI3 knockdown in the doxycycline-treated shEDI3 cells at time of injection. **C** Representative luminescence images of mice (left panel) showing signal intensity and corresponding quantitative analysis of luminescence signal for HCC1954-luc shNEG (upper right panel) and shEDI3 (lower right panel) normalized to T0. Data represent results of five mice per condition. Box plots: horizontal line, median; box, 25th–75th percentiles; whiskers, min to max (*p < 0.05; **p < 0.01)

To begin investigating if EDI3 influences metastasis formation in mice, an experimental model of lung metastasis was used. For this purpose, HCC1954-luc shNEG and shEDI3 cells were pre-treated with doxycycline for 72 h (Fig. 3A), EDI3 silencing was confirmed on the protein level (Fig. 3B), and cells were injected into the tail vein of doxycycline-treated and untreated CD1 nude mice. In vivo bioluminescence imaging revealed that silencing EDI3 in the HCC1954-luc shEDI3 cells with doxycycline significantly reduced the luminescence signal in the lungs at one, two- and four-weeks post injection compared to the non-induced shEDI3 cells (Fig. 3C). This reduction was not due to doxycycline as the luminescent signal was stronger in the lungs of mice injected with doxycycline-treated HCC1954-luc shNEG cells compared to the non-induced shNEG cells (Fig. 3C). However, over time the luminescence signal in the lungs became weaker in all mice until it was no longer detectable (Fig. 3C, bottom right panel; Additional file 11: Suppl. Figure S8A). We continued to monitor the mice for an additional nine weeks (15 weeks in total) to ascertain whether there were possible micrometastases that would eventually grow but observed no further signal; we also saw no visible macroscopic tumors on the lung surface when the mice were sacrificed (data not shown). Of note, we could measure luminescent signals in our HCC1954-luc cells even after 15 weeks in culture (Additional file 11: Suppl. Figure S8B) in the absence of antibiotic G418 which is needed for selection and maintenance of positively transduced clones, suggesting that the eventual loss of luminescent signal in vivo was not due to a loss in luciferase activity in the implanted tumor cells, but that the tumor cells may have eventually died.

**Fig. 4:**
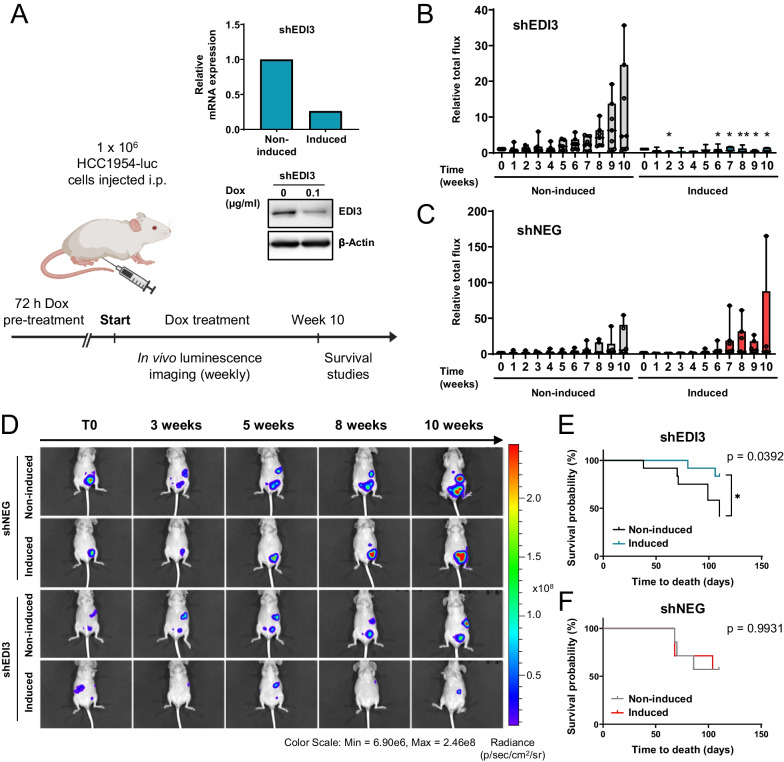
Silencing EDI3 in HCC1954 reduces formation of peritoneal metastasis in mice and improves survival. **A** Schematic illustration of the experimental plan. Luciferase-expressing HCC1954 shNEG and shEDI3 cells were induced with doxycycline for 72 h. EDI3 mRNA expression and corresponding Western blot showing reduced EDI3 expression in the HCC1954-luc shEDI3 cells at time of injection. Doxycycline-treated (induced) and non-treated (non-induced) cells were injected into the peritoneum of doxycycline pre-treated and untreated CD1 nude mice, respectively. Doxycycline was administered to the mice by a 625 mg/kg doxycycline containing diet (Ssniff) ad libitum. Luminescence signal was measured over ten weeks and the time of survival was recorded. Quantitative analysis of luminescence signal for **B** shEDI3 and **C** shNEG normalized to T0. **D** Corresponding representative luminescence images of mice. Survival of mice was observed for 15 weeks after intraperitoneal injection with **E** shEDI3 and **F** shNEG cells. Data in **B** and **C** represent seven mice per condition. Data in **E** and **F** represent 12 (shEDI3) or seven (shNEG) mice per condition. Box plots: horizontal line, median; box, 25th–75th percentiles; whiskers, min to max. Kaplan–Meier curves: *p* values were determined by log-rank test (*p < 0.05; **p < 0.01)

HCC1954 cells were reported to successfully form metastases in peritoneal organs and grow aggressively when injected into the peritoneum of immunodeficient mice [43]. This model was therefore used to investigate if silencing EDI3 reduces the formation of peritoneal metastasis. Both HCC1954-luc shNEG and shEDI3 cells were pretreated with doxycycline, decreased mRNA and protein levels were confirmed, and cells were injected intraperitoneally into doxycycline-treated and untreated control mice (Figure 4A). In vivo bioluminescence imaging revealed that the signal intensities in mice injected with non-induced shNEG and shEDI3 cells increased over time, confirming metastasis growth in this peritoneal metastasis model (Fig. 4B–D). Importantly, silencing EDI3 resulted in significantly less luminescence signal compared to non-induced shEDI3 cells (Fig. 4B). In contrast, doxycycline treatment of mice injected with HCC1954-luc shNEG cells did not decrease luminescence (Fig. 4C), showing that doxycycline itself had no reducing effect on metastasis growth.

### EDI3 knockdown improves survival

Since in vivo imaging demonstrated that the peritoneal metastasis burden, represented by the detected luminescence signal, was lower when EDI3 was silenced, we investigated if EDI3 expression influenced survival. CD1 nude mice injected intraperitoneally with doxycycline-induced and non-induced HCC1954-luc shNEG and shEDI3 cells were observed for 15 weeks (Fig. 4), with confirmation that tumor cells, as indicated by the luminescent signal, was still present at 13 weeks (Additional file 11: Figure S8C). Interestingly, the mice survived significantly longer when EDI3 was silenced (Fig. 4E). More specifically, only 16.7% of these mice died within the first 15 weeks after injection, compared to the 58.3% that were injected with non-induced shEDI3 cells. Injection of both induced and non-induced shNEG cells resulted in a probability of survival of 42.9% at 15 weeks (Fig. 4F), thereby excluding an influence of doxycycline treatment itself.

### Lower metastatic burden upon EDI3 knockdown

**Fig. 5:**
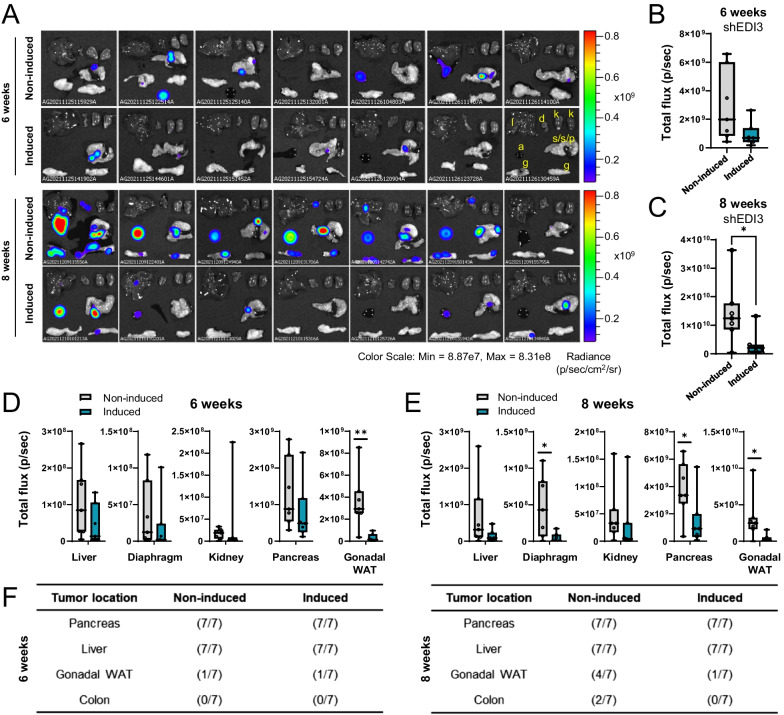
EDI3 knockdown in HCC1954-luc cells reduces metastatic burden. **A** Ex vivo luminescence imaging of organs and 100 μl ascites fluid (if present) with corresponding quantitative analysis **B** six and **C** eight weeks after intraperitoneal injection of doxycycline-induced and non-induced HCC1954-luc shEDI3 cells. Quantitative analysis of luminescence signal analyzed per organ after **D** six and **E** eight weeks. **F** Tables representing the ratio of mice positive for tumors in a specific organ compared to all mice six (left panel) and eight (right panel) weeks after injection. Data represent results of seven mice per condition. Box plots: horizontal line, median; box, 25th–75th percentiles; whiskers, min to max (*p < 0.05; **p < 0.01). l, liver; d, diaphragm; k, kidney; s/s/p, complex of spleen, stomach, and pancreas; a, ascites fluid; g, gonadal white adipose tissue (WAT)

The results obtained thus far showed that silencing EDI3 led to a reduction in luminescence in vivo after intraperitoneal injection of HCC1954 cells, indicating decreased metastasis formation. In a subsequent step, we wanted to determine the localization and size of the tumors. Therefore, organs were collected from mice six and eight weeks after intraperitoneal injection of doxycycline-induced and non-induced HCC1954-luc shEDI3 cells. Analysis of EDI3 mRNA expression in the cells at the time of injection revealed a 65% knockdown, which was confirmed on the protein level (Additional file 12: Suppl. Figure S9A-B). On the day of collection, luminescence intensities of the entire mouse were first measured in vivo, demonstrating reduced signal in mice injected with induced shEDI3 cells (Additional file 12: Suppl. Figure S9C), which was consistent with the previous experiment (Fig. 4). Mice were then sacrificed and ascites fluid, if present, was extracted before organs (liver, diaphragm, kidneys, complex of spleen/stomach/pancreas, and gonadal white adipose tissue [WAT]) were collected for ex vivo imaging. In general, we observed a reduction in the total luminescence signal in the organs of mice injected with induced HCC1954-luc shEDI3 cells compared to non-induced HCC1954-luc shEDI3 cells, which reached significance at eight weeks (Fig. 5A–C), thus agreeing with the whole mouse in vivo imaging results (Additional file 12: Suppl. Figure S9C).

To elucidate if EDI3 influences metastatic organotropism, we then analyzed the ex vivo luminescence data for each organ separately. After six weeks, the strongest signal was detected in the pancreas while the gonadal WAT was found to be the second most common site of metastasis of the HCC1954 cells (Fig. 5D). For both locations, the luminescence signal was lower when EDI3 was silenced, which was significant for the gonadal WAT. In addition to the elevated signals observed in pancreas and gonadal WAT at eight weeks, increased luminescence was also detected in liver and diaphragm of mice injected with non-induced shEDI3 cells (Fig. 5E). Importantly, silencing EDI3 significantly reduced the metastatic burden in the diaphragm, pancreas, and gonadal WAT, as well as the liver, although the latter did not reach significance. No difference in luminescence was measured in the kidneys. Altogether, our results show that while silencing EDI3 did not prevent metastases or alter metastatic organotropism (Fig. 5F), there was reduced tumor burden in the organs where tumors were present.

### Silencing EDI3 alters metabolite levels in tumors

**Fig. 6:**
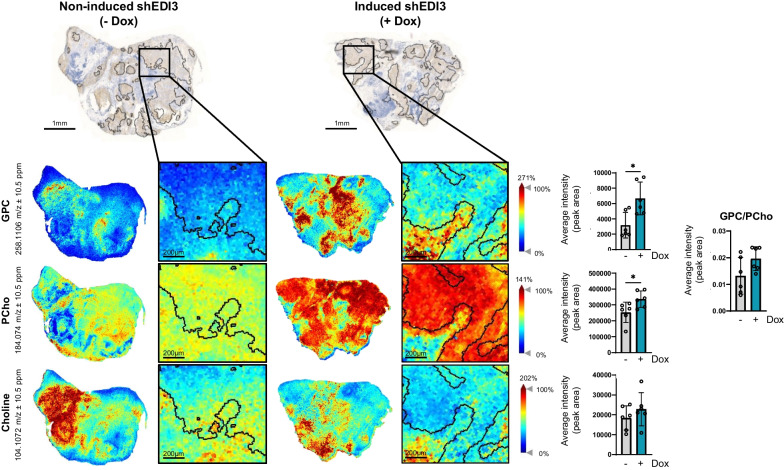
MALDI-MSI reveals altered GPC and PCho levels in tumors with silenced EDI3 expression. Representative images of HER2 staining and MALDI mass spectrometry imaging of tumors dissected from pancreas of mice eight weeks after intraperitoneal injection of non-induced (-Dox) and doxycycline-induced (+ Dox) HCC1954-luc shEDI3 with corresponding quantification of glycerophosphocholine (GPC), phosphocholine (PCho) and choline levels within the defined tumor regions (black lines), with the GPC/PCho ratio also provided. Scale bars represent 1 mm and 200 µm (zoomed). Values represent mean ± SD for six tumors per condition (*p < 0.05)

Our in vitro analyses demonstrated that silencing EDI3 in HCC1954 cells led to reduced anoikis resistance as well as higher levels of GPC and an increased GPC/PCho ratio. To study whether the decreased metastatic burden because of EDI3 knockdown is linked to similar metabolic changes in vivo, we next analyzed excised macroscopic tumors that were retrieved after intraperitoneal injection of doxycycline-induced and non-induced HCC1954-luc shEDI3 cells. The small size of the tumor nodules made dissecting tumors from the surrounding tissue challenging and therefore we were only able to obtain sufficient material from the pancreas of all mice collected at eight weeks. HER2 staining revealed that the tissue specimens were still heterogenous, consisting of both tumor cells and surrounding stroma (Fig. 6). Therefore, MALDI mass spectrometry imaging (MALDI-MSI), was used to spatially visualize and quantify metabolite levels in the tumor areas specifically. From the collected pancreas tumors, six per condition (non-induced or induced) passed quality control for MALDI-MSI and were subsequently analyzed. The obtained results show that GPC and phosphocholine were significantly increased in the tumor regions when EDI3 was silenced by doxycycline induction (Fig. 6), thus confirming the in vitro findings. Furthermore, although not significant, GPC/PCho ratio was increased in EDI3 silenced tumors. Similar to the in vitro results with HCC1954, we observed no significant alterations in choline levels.

### EDI3 knockdown reduces ascites formation

**Fig. 7:**
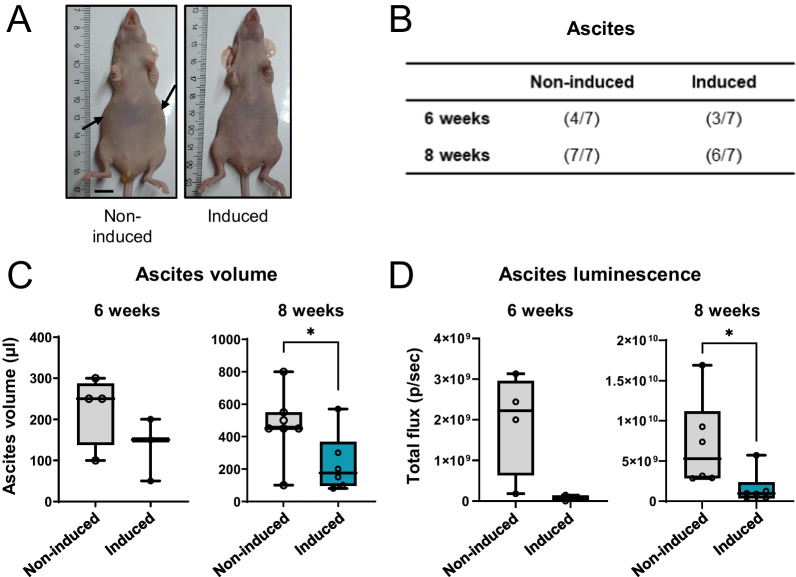
EDI3 knockdown in HCC1954-luc cells reduces ascites formation. **A** Representative photographs of mice with (left panel) and without (right panel) ascites. Swelling caused by ascites is indicated using arrows. Scale bar represents 1 cm. **B** Table representing the ratio of mice positive for ascites compared to all mice. **C** Ascites volume measured in mice. **D** Quantitative analysis of luminescence signal detected in 100 μl ascites fluid by ex vivo imaging. Data represent results of seven mice per condition. ‘Non-induced’ are mice injected intraperitoneally with HCC1954-luc shEDI3 cells not treated with doxycycline; ‘induced’ are mice injected with doxycycline-treated HCC1954-luc shEDI3 cells and fed a 625 mg/kg doxycycline containing diet (Ssniff) ad libitum for the duration of the experiment. Box plots: horizontal line, median; box, 25th–75th percentiles; whiskers, min to max (*p < 0.05)

Malignant ascites is the pathological accumulation of excessive fluid within the peritoneal cavity, which is caused by tumors that originate in or metastasize to the abdomen. The most common primary tumor leading to ascites is ovarian cancer, but also primary breast cancer was reported to cause peritoneal carcinomatosis and ascites [44–46]. To investigate if EDI3 expression influences ascites production, the fluid from ascites-bearing mice, as indicative by the more rounded abdomen (Fig. 7A) was collected six and eight weeks after intraperitoneal injection of HCC1954-luc shEDI3 cells. Doxycycline-induced EDI3 silencing in these cells had no influence on the prevalence of ascites (Fig. 7B) but resulted in reduced ascites volume compared to non-induced controls, which reached significance at eight weeks (Fig. 7C). Moreover, bioluminescence imaging of 100 µl ascites fluid revealed lower signal intensities when EDI3 was silenced, which also reached significance at eight weeks (Fig. 7D). The observed reduction in luminescence indicates that the ascites fluid contained less viable cancer cells.

## Discussion

Metastasis remains the main cause of cancer morbidity and mortality, and consequently, it is critical to understand the mechanisms that regulate metastasis, as they may lead to the identification of metastasis-promoting genes that serve as potential treatment targets. In the present work, we demonstrate that inducibly silencing the glycerophosphodiesterase EDI3 in ER-HER2+ HCC1954 breast cancer cells reduces the tumor burden subsequent to tail vein or intraperitoneal tumor cell injection using in vivo luminescence imaging in two mouse models of experimental metastasis. Further analysis into the latter peritoneal metastasis model indicated that mice injected with EDI3-silenced cells develop less ascites and survive longer. In vitro analyses using the same inducible cell model demonstrated that silencing EDI3 reduces adhesion and anoikis resistance, which was confirmed in a second ER-HER2+ cell line, SUM190PT.

The metastatic process includes a series of steps where cancer cells escape the primary tumor, invade the surrounding extracellular matrix, intravasate into the bloodstream, disseminate via the circulation, extravasate from the bloodstream, and colonize distant organs [47]. However, the mechanism by which EDI3 influences metastasis is currently unknown. Our early work in HER2- cell lines, MCF7 and MDA-MB-231 demonstrated that silencing EDI3 reduces cell migration [11, 27], a key feature acquired by cancer cells that enables them to leave the primary tumor and reach blood vessels through which they intravasate. Surprisingly, in the present study, silencing EDI3 in HCC1954 and SUM190PT cells had no impact on cell migration, but instead significantly reduced anoikis resistance in both cell lines; cell viability as shown in the present work for SUM190PT cells and previously for the HCC1954 [6]; and finally adhesion in the HCC1954 cell line.

To survive the transit through the lymphovascular system, cancer cells acquire resistance to anoikis, a form of cell death that occurs when cells become detached from the extracellular matrix and neighboring cells. Pathways and processes regulating anoikis resistance include metabolic networks, epithelial to mesenchymal transition pathways, adhesion proteins, extracellular pH, hypoxia, and reactive oxidative species [48]. Other studies have reported that increased pro-survival signaling, such as the overexpression of growth factor receptors or upregulation of PI3K/Akt signaling helps cells avoid anoikis by inhibiting apoptotic pathways [49–51]. With respect to alterations in metabolic pathways, changes in glucose [52–54] and fatty acid [55] metabolism have been shown to support anoikis resistance in cancer. To gain insight into how EDI3 mediated metabolism may be functionally associated with anoikis resistance and metastasis, we analyzed metabolic changes after EDI3 silencing. Interestingly, spatial analysis of excised pancreatic metastases by MALDI-MSI demonstrated increased levels of EDI3’s metabolic substrate GPC upon EDI3 silencing. A consistent increase in GPC levels was also observed in HCC1954 and SUM190PT, as well as in SKBR3 cells in response to EDI3 knockdown, indicating that EDI3 is indispensable for the breakdown of this intermediate in phosphatidylcholine (PtdCho) metabolism. The inability to metabolize GPC further may limit the availability of choline and G3P to produce membrane phospholipids that are needed to promote growth and metastasis. However, the direct link between higher GPC and decreased metastasis needs to be confirmed experimentally in further studies. Several previous studies have reported altered GPC levels in breast cancer. For instance, comparing breast cancer tissue with adjacent non-involved tissue, Sitter et al. reported a significantly lower GPC/PCho ratio in the tumors [24]. The same group also reported higher GPC levels in tumor tissue from patients with better prognosis five years post-surgery, although the difference was not statistically significant [56]. In contrast, other previously published findings suggest that high GPC levels are indicative of a more aggressive phenotype. For example, comparing metabolite levels in pre- versus post-treatment biopsies in breast cancer patients treated with neoadjuvant chemotherapy revealed significantly reduced GPC levels post-treatment in patients with partial response, which was not significant for patients with stable disease [57]. In the same study, GPC was also reduced post-treatment in survivors but not in non-survivors. Another study by the same group demonstrated reduced levels of GPC, PCho and tCho in patients who survived longer than five years, comparing post- versus pre-treatment specimens [58]. Tumors of the basal-like molecular subtypes, which is considered highly aggressive, was also shown to have a particularly high GPC/PCho ratio in patient-derived animal tumor models, which was corroborated by the analysis of tumor tissue from patients with triple-negative breast cancer [59]. None of the above studies specifically focused on HER2 + breast cancer and while several hypotheses have been put forth to explain this aberrant choline metabolism in more aggressive tumors (to provide cancer cells with more energy, membrane components for proliferation, restructure membranes to facilitate migration, provide bioactive signaling lipids, or to reshape and prime the microenvironment for invasion), the underlying mechanism is still to be elucidated.

The specific mechanism by which EDI3 influences anoikis resistance in ER-HER2+ cells is currently also not known, and there are no published reports linking EDI3 or GPC with anoikis. One possibility may be that deregulation of metabolism in response to EDI3 knockdown compromises membrane structure and stability leading to reduced viability and anoikis resistance. Upstream of EDI3, the production of PtdCho occurs via several different pathways. In the Lands cycle [60], fatty acid chains are removed from PtdCho by phospholipase A (PLA) enzymes to produce lysophosphatidylcholine (LPC), and fatty acids are added back to LPC via lysophosphatidylcholine acyltransferases to once more produce PtdCho. Alternatively, LPC can be further metabolized by PLA that removes the second fatty acid chain to produce GPC. A recent study showed that adding LPC to cells was enough to induce anoikis, and the authors provided evidence that increased anoikis was due to changes in membrane structure and cytoskeletal disorganization that reduced cell–cell contact and cell-extracellular matrix contact leading to death; however, the fate of LPC was not investigated [61]. PtdCho is also produced via the Kennedy pathway [62]—the only pathway known for the de novo synthesis of PtdCho—where the rate limiting step is catalyzed by CTP:phosphocholine cytidylyltransferase α (CCTα; PCYT1A) that makes CDP-Cho from PCho, prior to the final generation of PtdCho by DAG:CDP-choline cholinephosphotransferase using CDP-Cho and diacylglycerol (DAG). Moreover, an earlier publication showed that a shift in the levels of choline metabolites upon altering the expression of CCTα reduced anoikis resistance [63], with the authors proposing that high CCTα expression increased the cell’s production of PtdCho which facilitates anchorage-independent growth and supports malignant transformation. Many of the enzymes involved in choline and phospholipid metabolism are regulated by oncogenic signaling, such as CCTα, CHKA, PLA, phospholipase C and phospholipase D by RAS and its effectors Raf and PI3K as previously reviewed [18]. Similar signaling pathways have been linked to anoikis resistance [64], as well as transcription factors, like HIF1 that is activated via signaling downstream of HER2 to regulate anoikis resistance [51]. To date, there is still little known about the regulation of EDI3. In our recent work, we showed that EDI3 expression is regulated via PI3K/mTORC/GSK3β signaling downstream of HER2 [6]. Thus, while the previously published findings on choline metabolic enzymes do provide some clues as to the possible role of EDI3 in anoikis resistance, they remain speculative and further experiments are needed to provide mechanistic insights.

In contrast to the consistently increased GPC levels observed in three cell lines, EDI3 silencing elicited cell line specific changes in the levels of EDI3’s direct metabolic products (choline and G3P) and metabolites downstream of both. Since there does not appear to be a common metabolic pattern upon EDI3 silencing in the three studied ER-HER2+ cell lines, we can only currently speculate how EDI3 may be regulating these downstream processes, until further experiments are performed. A last factor to consider when aiming to understand how EDI3 may influence cancer-related endpoint is that in addition to its enzymatic glycerophosphodiesterase domain, EDI3 also contains a carbohydrate binding domain that remains largely unexplored, which we have shown in unpublished work to bind glycogen. In addition to being an energy source, glycogen also plays a critical role in redox regulation, cell differentiation, signaling, and stemness [65]; therefore, it cannot be excluded that non-enzymatic functions of EDI3 are also important for the observed phenotypic changes.

The in vitro and in vivo models used to study EDI3 in the present work have both advantages and limitations. With the inducible system, EDI3 expression can be regulated with respect to how much and at what point in an experiment it is silenced. However, a drawback of using doxycycline to inducibly silence EDI3 expression is that doxycycline on its own can influence cellular functions. For example, cell adhesion of smooth muscle cells has been reported to be enhanced by doxycycline [66]. In the present study, doxycycline increased the adhesion of HCC1954 control cells in vitro and enhanced lung metastases in mice. However, since the doxycycline-induced EDI3 knockdown led to the opposite effect, namely a significant reduction of HCC1954 adhesion and lung metastasis formation, this observation can still be interpreted because of reduced EDI3 expression. Nevertheless, it underlines the importance of doxycycline controls that were included in all experiments of the present study. At present, there is no EDI3-specific inhibitor available, which would circumvent the potential drawbacks related with silencing EDI3. But, based on our previous work that the general phosphodiesterase inhibitor, dipyridamole inhibits EDI3 activity, viability and tumor growth of ER-HER2+ cells in vitro and in vivo [6], in addition to identifying a specific small molecule inhibitor, we will also investigate existing drugs, for example other phosphodiesterase inhibitors to determine if already available, pharmacologically tested compounds can be repurposed to inhibit EDI3 and tumor growth and metastasis in vivo.

The experimental metastasis models used in the present work also have their advantages and limitations. By circumventing the early steps of metastasis, these models focus on the later stages of the process, namely extravasation and colonization of specific organ or organs [67, 68]. The advantage of these models is that they can be used to directly test the effect of targeting specific genes, like EDI3, on late-stage metastasis using genetic and pharmacologic interventions. Silencing EDI3 reduced the metastatic burden, irrespective of the route of application of cells. While it should be acknowledged that the intraperitoneal model may not be the most obvious choice to study breast cancer metastasis, intraperitoneal spread has been reported in late-stage metastatic disease, also in breast cancer [44, 69–71]. In addition, the data from the intraperitoneal model do support our overall conclusion that targeting EDI3 reduces metastasis. This conclusion is also supported by the findings in the lung metastasis model at the early timepoints studied where a significant decrease in luminescence was seen in the lungs of mice implanted with doxycycline-induced shEDI3 cells compared to those injected with non-induced cells. We did, however, also observe a reduction in luminescence in the induced and non-induced shNEG, as well as in the non-induced shEDI3 bearing mice over time. It was unlikely that this loss in signal was due to impaired luciferase activity or loss of the luciferase plasmid, since the luminescent signal remained stable in our HCC1954-luc cells for up to 15 weeks in culture (Additional file 11: Suppl. Figure S8B), and we also still observed in vivo luminescence signals in our peritoneal metastasis model after 13 weeks (Additional file 11: Suppl. Figure S8C).

Finally, despite the reproducibility and ease-of-use of experimental metastases models, there are also some general limitations, including the inability to recapitulate early metastatic events due to the absence of a primary tumor [67]. Increasing evidence suggests that the primary tumor may directly influence metastasis outcome [72]. For example, primary tumors have been reported to release exosomes-containing proteins, cytokines, and microRNAs into the circulation, which then travel to secondary sites, reshaping them to form pre-metastatic niches that enable homing and survival of circulating tumor cells [73, 74]. Moreover, the microenvironment surrounding the primary tumor, e.g., hypoxia, may alter gene expression in tumor cells that may then influence both their dissemination and dormancy fates and in turn therapy response [75]. Thus, subsequent studies will investigate EDI3’s role in metastasis utilizing orthotopic models that better recapitulate the complete metastasis process.

## Conclusions

The present study shows that silencing the choline-producing glycerophosphodiesterase EDI3 alters intracellular levels of key glycerophospholipids, reduces colony formation, cell adhesion, and anoikis-resistance in vitro, as well as decreases the metastatic burden in two experimental metastasis models of ER-HER2+ breast cancer in vivo. Altogether, EDI3 may be a potential target to prevent or reduce metastasis formation that warrants further exploration.

### Supplementary Information

Additional file 1. Supplementary Table S1: List of used reagents including (A) shRNA and siRNA oligos, (B) QuantiTect primer assays and (C) antibodies.

Additional file 2. Supplementary Material and Methods: Detailed description of material and methods, including EDI3 silencing, cell culture conditions of SUM190PT, viability of adherent cells and metabolite analysis

Additional file 3: Supplementary Table S2: Transition data for measurement of metabolites

Additional file 4. Supplementary Figure S1: MALDI-MSI reveals that the mass of 706.54 m/z correlates with HER2 staining. Representative images of HER2 staining (left) and the mass of 706.54 m/z measured using MALDI-MSI (middle) of tumors dissected from pancreas of CD1 nude mice eight weeks after intraperitoneal injection of doxycycline-induced and non-induced HCC1954-luc shEDI3 cells. The mass of 706.54 m/z (candidate molecule PC 30:0) correlated in a large part with HER2 staining and was used to generate tumor-cell-containing areas-of-interest (right). Scale bars represent 1 mm

Additional file 5. Supplementary Figure S2: Silencing EDI3 alters intracellular choline metabolite levels in ER-HER2+ breast cancer cells. Intracellular levels of EDI3’s substrate glycerophosphocholine (GPC), as well as EDI3 products choline and G3P (also shown in Figure 1) and choline products phosphocholine (PCho) and betaine measured using LC-MS/MS 72 h after silencing EDI3 (A) inducibly with shRNA in HCC1954 or transiently with siRNA in (B) SUM190PT and (C) SKBR3 breast cancer cells. Metabolite levels were determined by calculating the ratios of the integrated peaks of the endogenous metabolites and the internal standards. Quantities of metabolites were normalized to cell number and presented relative to negative control (shNEG or siNEG). Data represent mean ± SD from at least five technical replicates (wells from a six-well plate) (*p < 0.05; **p < 0.01; ***p < 0.001; ****p < 0.0001)

Additional file 6. Supplementary Figure S3: EDI3 knockdown in SUM190PT and SKBR3 cells used for metabolite analysis. EDI3 mRNA expression and Western blots with corresponding quantification showing EDI3 protein expression after silencing EDI3 in (A) SUM190PT and (B) SKBR3 breast cancer cells compared with cells transfected with scrambled siRNA (siNEG #1). Replicates of these cells were used for the analysis of intracellular choline metabolites and lipids by LC-MS/MS. FM, full media control

Additional file 7. Supplementary Figure S4: Silencing EDI3 alters glycerophospholipid levels in ER-HER2+ breast cancer cells. Intracellular levels of different species of LPA, PA, LPC, PtdCho, LPS, PS, LPG, PG, LPE, PE, LPI and PI were measured using LC-MS/MS 72 h after silencing EDI3 either inducibly with shRNA in HCC1954 or transiently with siRNA in SUM190PT and SKBR3 breast cancer cells. Metabolite levels were determined by calculating the ratios of the integrated peaks of the endogenous metabolites and the internal standards. Quantities of metabolites were normalized to cell number and presented relative to negative control (shNEG or siNEG). Data represent mean ± SD from at least five technical replicates (wells from a six-well plate) from one or two independent experiments (*p < 0.05; **p < 0.01; ***p < 0.001; ****p < 0.0001). LPA, lysophosphatidic acid; PA, phosphatidic acid; LPC, lysophosphatidylcholine; PtdCho, phosphatidylcholine; LPS, lysophosphatidylserine; PS, phosphatidylserine; LPG, lysophosphatidylglycerol; PG, phosphatidylglycerol; LPE, lysophosphatidylethanolamine; PE, phosphatidylethanolamine; LPI, lysophosphatidylinositol; PI, phosphatidylinositol

Additional file 8. Supplementary Figure S5: EDI3 silencing using siRNA does not influence migration in ER-HER2+ HCC1954 cells. Percentage of wound closure relative to negative control (siNEG) after silencing EDI3 with three siRNA oligos targeting different exons in HCC1954 cells. Values in graph represent mean ± SD from two independent experiments. FM, full media control

Additional file 9. Supplementary Figure S6: Replenishing cell media with fresh doxycycline does not additionally affect EDI3 expression nor colony formation. Non-induced HCC1954_Luc shEDI3 cells were plated for colony formation assay in media containing 0 µg/ml or 0.1 µg/ml doxycycline. Media was either not changed (-mc) over the assay period of 14 days or media was replenished +/- fresh doxycycline every 3 days (+mc). A) EDI3 mRNA expression after 14 days of colony formation assay both with and without media change. B) Representative images (left) and corresponding quantification of colony number (middle) and size (right). Values in graphs represent mean ± SD from three independent experiments (*p < 0.05; **p < 0.01; ns, not significant)

Additional file 10. Supplementary Figure S7: Silencing EDI3 in SUM190PT cells using siRNA reduces resistance to anoikis and viability in adherent cells. (A) EDI3 mRNA and (B) protein expression after silencing EDI3 in SUM190PT cells compared with cells transfected with two different scrambled siRNA (siNEG #1 and #2). Viability in RFU relative to negative control measured in (C) non-adherent cells 48 h after plating on a poly-HEMA matrix or in (D) adherent cells 96 h after plating. Data represent mean ± SD from three independent experiments (*p < 0.05; ***p < 0.001). FM, full media control; RFU, relative fluorescence units

Additional file 11: Supplementary Figure S8: Luminescence signals detected in lungs after tail vein injection of HCC1954-luc cells decrease over time. (A) Luciferase-expressing HCC1954 shNEG and shEDI3 cells were treated with doxycycline for 72 h, followed by tail vein injection into doxycycline pre-treated and untreated CD1 nude mice, respectively. In contrast to the images of the same mice presented in Figure 3, where the luminescent signals from induced and non-induced mice were compared to one another each week, here, luminescence signals are shown on one scale to visualize how the signal intensity declines over the period of six weeks from time 0. Doxycycline was administered to the mice by a 625 mg/kg doxycycline containing diet (Ssniff) ad libitum. Representative luminescence images of five mice per condition are shown. (B) Luminescence signal remains stable in HCC1954-luc cells for 15 weeks, even in the absence of antibiotic G418 which is needed for selection and maintenance of positively transduced clones. This was an important control for the in vivo experiments, as the cells were no longer under the selection pressure of G418 once injected into mice. Luciferase assay was performed over time with HCC1954-luc cells cultured +/- G418 for up to 15 weeks. Measurements represent mean ± SD of three technical replicates. (C) Luminescence signal 13 weeks after intraperitoneal (IP) injection of HCC1954-luc shEDI3 in CD1 nude mice confirms the presence of tumor cells in mice that were used to assess survival time. Luminescence was measured 3 min after administration of luciferin

Additional file 12. Supplementary Figure S9: In vivo bioluminescence imaging confirms reduced peritoneal metastases in mice prior to organ collection. HCC1954-luc shEDI3 cells were induced with doxycycline for 72 h. Induced and non-induced cells were injected into the peritoneum of doxycycline pre-treated and untreated CD1 nude mice, respectively. Doxycycline was administered to the mice by a diet containing 625 mg/kg doxycycline (Ssniff) ad libitum. (A) EDI3 mRNA expression and (B) corresponding Western blot showing EDI3 protein expression in the cells at time of injection. (C) In vivo luminescence images acquired six and eight weeks after injection (left) and corresponding quantitative analysis of luminescence signal normalized to T0 (right). Data in (C) represent seven mice per condition. Box plots: horizontal line, median; box, 25th-75th percentiles; whiskers, min to max (*p < 0.05)

## Data Availability

All data generated or analyzed during this study are available from the corresponding author on reasonable request.

## Abbreviations

CBM20: Carbohydrate binding moiety 20
CCD: Charge-coupled device
CCTα: CTP:phospho-choline cytidylyltransferase α
CDP-Cho: Cytidine diphosphate choline
CDP-DAG: Cytidine diphosphate diacylglycerol
DAG: Diacylglycerol
DHAP: Dihydroxyacetone phosphate
EDI3: Endometrial carcinoma differential 3
ER: Estrogen receptor
G3P: Glycerol-3-phosphate
GDE: Glycerophosphodiesterase
GPC: Glycerophosphocholine
GPCPD1: Glycerophosphocholine phosphodiesterase 1
HER2: Human epidermal growth factor receptor 2
LC-MS: Liquid chromatography-mass spectrometry
LPA: Lysophosphatidic acid
LPC: Lysophosphatidylcholine
LPE: Lysophosphatidylethanolamine
LPG: Lysophosphatidylglycerol
LPI: Lysophosphatidylinositol
LPS: Lysophosphatidylserine
MALDI: Matrix-assisted laser desorption ionization
MSI: Mass spectrometry imaging
PA: Phosphatidic acid
PCho: Phosphocholine
PE: Phosphatidylethanolamine
PG: Phosphatidylglycerol
PGP: Phosphatidylglycerol phosphate
PI: Phosphatidylinositol
PI3K: Phosphatidylinositol 3-kinase
PLA: Phospholipase A
Poly-HEMA: Poly(2-hydroxyethyl methacrylate)
PS: Phosphatidylserine
PtdCho: Phosphatidylcholine
RFU: Relative fluorescence units
siRNA: Silencing RNA
TIC: Total ion current
WAT: White adipose tissue
