# Supplementary material for: EDI3 knockdown in ER-HER2+ breast cancer cells reduces tumor burden and improves survival in two mouse models of experimental metastasis
Source: Breast Cancer Res. 2024 May 30;26:87. doi: 10.1186/s13058-024-01849-y (PMC11138102; doi:10.1186/s13058-024-01849-y)
Supplement: Supplementary file 1 — Additional file 1. Supplementary Table S1: List of used reagents including (A) shRNA and siRNA oligos, (B) QuantiTect primer assays and (C) antibodies. [file 13058_2024_1849_MOESM1_ESM.docx]

**Supplementary Table S1.** List of used reagents including (A) shRNA and siRNA oligos, (B) QuantiTect primer assays and (C) antibodies.

**A. List of oligos (shRNA oligos from Dharmacon; siRNA oligos from Thermo Scientific)**

| Gene | Product | Article number | Sequence |
| --- | --- | --- | --- |
| shEDI3 #1 | SMARTvector^TM^ | V3SH7669-228754853 | CCAGAAGATGTAGGGTTTA |
| shEDI3 #2 | SMARTvector^TM^ | V3SH7669-230425247 | GGAGTTAATGGTCTAATTT |
| shNEG | SMARTvector^TM^ | VSC6571 | Not provided |
| siEDI3 #1 | Stealth RNAi™ | 1299001-HSS125510 | GCUCACUCAUGUGACUGCACUGAAA |
| siEDI3 #2 | Silencer®-Select | 4392420-ID s32104 | CGAAAUUAUUAUUGACGAUtt |
| siEDI3 #3 | Silencer®-Select | 4392420-ID s32106 | GCGAAAUUAUUAUUGACGCtt |
| siEDI3 #4 | Stealth RNAi™ | 1299001-HSS125509 | GGCAAAGUGAGAGUUGACUAUAUAA |
| siEDI3 #5 | Silencer®-Select | 4392420-ID s32105 | GGAUGGUAACUUAUCAACAtt |
| Negative Control Low GC Duplex #2 | Stealth RNAi™ | 12935-110 | Not provided |

**B. List of QuantiTect primer assays (from Qiagen)**

| Gene | Primer assay | Article number |
| --- | --- | --- |
| GPCPD1 (EDI3) | Hs_GPCPD1_1_SG | QT00066598 |
| ACTB | Hs_ACTB_1_SG | QT00095431 |

**C. List of antibodies**

| Protein target | Company | Article number | Dilution |
| --- | --- | --- | --- |
| EDI3, clone 3B8G3 | AMS Bio | Custom Made | 1:1000 |
| β-actin (anti rabbit) | Cell Signaling | 4967S | 1:1000 |
| β-actin (anti mouse) | Sigma | A5316 | 1:3000 |
| Anti-Rabbit IgG | Cell Signaling | 7074S | 1:1000 |
| Anti-Mouse IgG | Cell Signaling | 7076S | 1:1000 |
| HER2 (anti rabbit) | Abcam | ab134182 | 1:2000 |
