# Supplementary material for: EDI3 knockdown in ER-HER2+ breast cancer cells reduces tumor burden and improves survival in two mouse models of experimental metastasis
Source: Breast Cancer Res. 2024 May 30;26:87. doi: 10.1186/s13058-024-01849-y (PMC11138102; doi:10.1186/s13058-024-01849-y)
Supplement: Supplementary file 2 — Additional file 2. Supplementary Material and Methods: Detailed description of material and methods, including EDI3 silencing, cell culture conditions of SUM190PT, viability of adherent cells and metabolite analysis [file 13058_2024_1849_MOESM2_ESM.docx]

**SUPPLEMENTARY MATERIAL & METHODS**

**EDI3 knockdown in ER-HER2+ breast cancer cells reduces the tumor burden and improves survival in two mouse models of metastasis**

Annika Glotzbach^1^, Katharina Rohlf^1^, Anastasia Gonscharow^1^, Simon Lüke^1^, Özlem Demirci^1,2^, Brigitte Begher-Tibbe^1^, Nina Overbeck^1^, Jörg Reinders^1^, Cristina Cadenas^1^, Jan G. Hengstler^1^, Karolina Edlund^1^, Rosemarie Marchan^1^

^1^Leibniz Research Centre for Working Environment and Human Factors at the TU Dortmund (IfADo), Dortmund, Germany

^2^Science Faculty, Department of Biology, Dicle University, Diyarbakır, Turkey

Corresponding author: Rosemarie Marchan, Leibniz Research Centre for Working Environment and Human Factors at the Technical University Dortmund (IfADo), Ardeystrasse 67, Dortmund D-44139, Germany. Phone: +49 2311084213; Fax: +49 231 1084 403

E-mail: marchan@ifado.de

**Cell culture conditions for SUM190PT and SKBR3**

Human breast cancer cell line SUM190PT was purchased from BioIVT and grown in RPMI1640 (stable Glutamine and 2.0 g/L NaHCO3, PAN, Biotech) supplemented with 10% fetal bovine serum (FBS, Thermo Fisher Scientific), 5 µg/ml human insuline (Santa Cruz Biotechnology) and 1 µg/ml Hydrocortison (Sigma-Aldrich). Human breast cancer cell line SKBR3 was purchased from the German Collection of Microorganisms and Cell Cultures (DSMZ) and grown in RPMI1640 (stable Glutamine and 2.0 g/L NaHCO3, PAN, Biotech) supplemented with 10% fetal bovine serum (FBS, Thermo Fisher Scientific). Cells were authenticated by DSMZ according to the ANSI/ATCC ASN-0002–2011 guidelines and were regularly tested for mycoplasma using the Venor® GeM Classic kit (Minerva Biolabs).

**EDI3 silencing**

To stably and inducibly silence EDI3, HCC1954-luc cells were transduced with the SMARTvector^TM^ lentiviral particles (Dharmacon) containing two shRNA oligos targeting different exons of EDI3 (Supplementary Table S1A) under the control of a Tet-On 3G tetracycline-inducible system, as well as a non-targeting scrambled shRNA control, as previously described [1]. To induce EDI3 knockdown, cells were treated with doxycycline (Sigma-Aldrich) as indicated for the different endpoints. For transient downregulation of EDI3 in HCC1954, SKBR3 or SUM190PT cells, siRNA oligos were brought into the cells by reverse transfection with Lipofectamine RNAiMax Reagent (Thermo Fisher Scientific) according to the manufacturer’s instruction. Briefly, 500 µl Opti-MEM, 20 nm siRNA Oligos and 5 µl Lipofectamine RNAiMax Reagent were combined in wells of a six-well plate followed by addition of 0.3 x 10^6^ cells for HCC1954, 0.4 x 10^6^ for SKBR3 or 0.6 x 10^6^ for SUM190PT in 2.5 ml antibiotic-free medium per well. Cells were incubated for 72 h to ensure knockdown at the RNA and protein level before they were used for subsequent experiments. To silence gene expression siRNA oligos targeting different exons of EDI3 were used while non-targeting siRNAs served as negative controls (all from Thermo Fisher Scientific). All siRNA sequences are displayed in Supplementary Table S1A.

**Viability of adherent cells**

SUM190PT cells were plated onto 6-well plates and transfected with siRNA oligos against EDI3 or non-targeting oligos as described above. Following 72 h transfection, cells were re-seeded in duplicates in 96 well plates at a density of 1.5 x 10^4^ cells per well. Media was changed 48 h after re-seeding. After 96 h, the amount of viable cells was determined using the CellTiter-Blue® Cell Viability Assay as described previously [1]. Briefly, cells were incubated with media supplemented with CellTiter-Blue (CTB) reagent (5:1) for 3.5 h. Fluorescence was measured using a plate reader (Infinite M200 Pro, Tecan) with a 560Ex/590Em filter set.

**Metabolite analysis**

To extract metabolites from cells, cell culture dishes with six technical replicates (wells from a six-well plate) were placed on ice, medium was aspirated, and cells were washed with ice cold 1x PBS thrice. After the last washing step cells were snap-frozen by placing the well plates on a thin layer of liquid nitrogen. Ice cold methanol spiked with internal standards was added to the wells. Cells were scraped, collected, and kept on ice. All extracts were stored at -80°C until further processing. Replicate wells for all conditions were used to determine the cell number per well with the CASY-TT cell counter. The extracted metabolite samples were fractionated using the simultaneous metabolite, protein, lipid extraction (SIMPLEX) protocol [2]. Briefly, 300 μl of cold methyl-tert-butyl-ester (MTBE) was added to the samples, followed by incubation for 1 h at 4°C under agitation. To induce phase separation, samples were mixed with 80 μl aqueous 0.1% ammonium formiate and centrifuged at 10,000 x g for 5 min. The upper phase (fraction 1), which contains most of the lipids, was collected, dried under nitrogen flow, and stored at -20°C until reconstitution. The lower phase was supplemented with 220 μl methanol and incubated for 2 h at -20 C to achieve complete protein precipitation followed by centrifugation at 21,000 x g for 5 min. The supernatant (fraction 2), containing choline metabolites as well as some lipids, was transferred into a new tube and evaporated to dryness. Fraction 1 was reconstituted using 40 μl 80% acetonitrile/20% methanol with 1 mM phosphoric acid while fraction 2 was solubilized in 40 μl methanol. Both fractions were measured in both positive and negative ion mode. LC-MS/MS-analysis of the lipids contained in both fractions was performed on a QTrap 5500 mass spectrometer (Sciex) operating in (scheduled) MRM-mode coupled to a LC20 UFLC system (Shimadzu). For this purpose, 1 μl per sample was injected into a 125 mm long, 3 mm PerfectSil Target 100 Sil column with 3 μm particle diameter. Samples were separated isocratically using 70% solvent A (acetonitrile) and 30% solvent B (50% methanol, 5 mM ammonium formiate, 0.1% formic acid) within 10 min. The settings are presented in Table 1. LC-MS/MS-analysis of the choline metabolites (fraction 2) was accomplished on a QExactive mass spectrometer (Thermo) directly coupled to a Vanquish Horizon UHPLC system (Thermo). One µl from each sample was injected into a 125 mm long, 3 mm I.D. Nucleoshell Bluebird RP18 column with 2.7 μm particle diameter and separated using a binary gradient (solvent A: 0.1% formic acid; solvent B: 0.1% formic acid in acetonitrile) at a flow rate of 400 μl/min (0-1.5 min: 0% B; 1,5-2.5 min: 0-80% B; 2.5-4.0 min: 80% B; 4-7 min: 0% B). The mass spectrometer was operated in positive mode using the parameters which are listed in Supplementary Table S2. All LC-MS/MS data were interpreted using the Skyline Daily software [3].

**References**

1. Keller M, Rohlf K, Glotzbach A, Leonhardt G, Lüke S, Derksen K, Demirci Ö, Göçener D, AlWahsh M, Lambert J *et al*: **Inhibiting the glycerophosphodiesterase EDI3 in ER-HER2+ breast cancer cells resistant to HER2-targeted therapy reduces viability and tumour growth**. *Journal of experimental & clinical cancer research : CR* 2023, **42**(1):25.

2. Coman C, Solari FA, Hentschel A, Sickmann A, Zahedi RP, Ahrends R: **Simultaneous Metabolite, Protein, Lipid Extraction (SIMPLEX): A Combinatorial Multimolecular Omics Approach for Systems Biology**. *Mol Cell Proteomics* 2016, **15**(4):1453-1466.

3. Adams KJ, Pratt B, Bose N, Dubois LG, St John-Williams L, Perrott KM, Ky K, Kapahi P, Sharma V, MacCoss MJ *et al*: **Skyline for Small Molecules: A Unifying Software Package for Quantitative Metabolomics**. *J Proteome Res* 2020, **19**(4):1447-1458.
