# Supplementary material for: EDI3 knockdown in ER-HER2+ breast cancer cells reduces tumor burden and improves survival in two mouse models of experimental metastasis
Source: Breast Cancer Res. 2024 May 30;26:87. doi: 10.1186/s13058-024-01849-y (PMC11138102; doi:10.1186/s13058-024-01849-y)
Supplement: Supplementary file 3 — Additional file 3: Supplementary Table S2: Transition data for measurement of metabolites [file 13058_2024_1849_MOESM3_ESM.docx]

**Supplementary Table S2.** Transition data for measurement of metabolites.

| Analyte | Q1 mass [Da] | Q3 mass [Da] | Mode |
| --- | --- | --- | --- |
| choline | 104.1 | 60.1 | positive |
| d9-choline | 113.2 | 69.1 | positive |
| phosphocholine | 184.1 | 86.1 | positive |
| d9-phosphocholine | 193.1 | 95.2 | positive |
| glycerophosphocholine | 258.1 | 125.0; 104.1; 86.1 | positive |
| d9-glycerophosphocholine | 267.2 | 113.2 | positive |
| betaine | 118.09 | 58.07 | positive |
| betaine | 118.09 | 59.07 | positive |
| d9-betaine | 127.14 | 66.14 | positive |
| d9-betaine | 127.14 | 68.13 | positive |
| glycerol-3-phosphate | 171.01 | 78.96 | negative |
| 13C3-glycerol-3-phosphate | 174.02 | 78.96 | negative |
| 16:0 LPA | 409.2 | 153.0 | negative |
| 18:0 LPA | 437.3 | 153.0 | negative |
| 18:1 LPA | 435.3 | 153.0 | negative |
| 17:1 LPA | 421.2 | 153.0 | negative |
| 32:0 PA | 647.5 | 255.2 | negative |
| 34:0 PA | 675.5 | 255.2 | negative |
| 34:1 PA | 673.5 | 255.2 | negative |
| 36:0 PA | 703.5 | 283.3 | negative |
| 36:1 PA | 701.5 | 283.3 | negative |
| 18:0-18:2 PA | 699.5 | 283.3 | negative |
| 18:1-18:1 PA | 699.5 | 281.2 | negative |
| 17:0-14:1 PA | 631.4 | 269.2 | negative |
| 16:0 LPC | 496.3 | 184.1 | positive |
| 16:1 LPC | 494.3 | 184.1 | positive |
| 18:0 LPC | 524.4 | 184.1 | positive |
| 18:1 LPC | 522.4 | 184.1 | positive |
| 18:2 LPC | 520.3 | 184.1 | positive |
| 18:3 LPC | 518.3 | 184.1 | positive |
| 20:4 LPC | 544.3 | 184.1 | positive |
| 17:1 LPC | 508.3 | 184.1 | positive |
| 32:0 PtdCho | 734.6 | 184.1 | positive |
| 34:0 PtdCho | 762.6 | 184.1 | positive |
| 34:1 PtdCho | 760.6 | 184.1 | positive |
| 34:2 PtdCho | 758.6 | 184.1 | positive |
| 36:0 PtdCho | 790.6 | 184.1 | positive |
| 36:1 PtdCho | 788.6 | 184.1 | positive |
| 36:2 PtdCho | 786.6 | 184.1 | positive |
| 17:0-14:1 PtdCho | 718.5 | 184.1 | positive |
| 16:0 LPS | 498.3 | 313.3 | positive |
| 16:1 LPS | 496.3 | 311.3 | positive |
| 18:0 LPS | 526.3 | 341.3 | positive |
| 18:1 LPS | 524.3 | 339.3 | positive |
| 18:2 LPS | 522.3 | 337.3 | positive |
| 17:1 LPS | 510.3 | 325.3 | positive |
| 32:0 PS | 736.5 | 551.5 | positive |
| 34:0 PS | 764.5 | 579.5 | positive |
| 34:1 PS | 762.5 | 577.5 | positive |
| 34:2 PS | 760.5 | 575.5 | positive |
| 36:0 PS | 792.6 | 607.6 | positive |
| 36:1 PS | 790.6 | 605.6 | positive |
| 36:2 PS | 788.5 | 603.5 | positive |
| 17:0-14:1 PS | 720.5 | 535.5 | positive |
| 16:0 LPG | 483.3 | 255.2 | negative |
| 18:0 LPG | 511.3 | 283.3 | negative |
| 18:1 LPG | 509.3 | 281.2 | negative |
| 18:2 LPG | 507.3 | 279.2 | negative |
| 17:1 LPG | 495.3 | 267.2 | negative |
| 32:0 PG | 721.5 | 255.2 | negative |
| 34:0 PG | 749.5 | 255.2 | negative |
| 34:1 PG | 747.5 | 255.2 | negative |
| 36:1 PG | 775.5 | 283.3 | negative |
| 18:1-18:1 PG | 773.5 | 281.2 | negative |
| 17:0-14:1 PG | 705.5 | 225.2 | negative |
| 16:0 LPE | 454.3 | 313.3 | positive |
| 16:1 LPE | 452.3 | 311.3 | positive |
| 18:0 LPE | 482.3 | 341.3 | positive |
| 18:1 LPE | 480.3 | 339.3 | positive |
| 18:2 LPE | 478.3 | 337.3 | positive |
| 18:3 LPE | 476.3 | 335.3 | positive |
| 20:4 LPE | 502.3 | 361.3 | positive |
| 17:1 LPE | 466.3 | 325.3 | positive |
| 32:0 PE | 692.5 | 551.5 | positive |
| 34:0 PE | 720.6 | 579.5 | positive |
| 34:1 PE | 718.5 | 577.5 | positive |
| 34:2 PE | 716.5 | 575.5 | positive |
| 36:0 PE | 748.6 | 607.6 | positive |
| 36:1 PE | 746.6 | 605.6 | positive |
| 36:2 PE | 744.6 | 603.5 | positive |
| 17:0-14:1 PE | 676.5 | 535.5 | positive |
| 16:0 LPI | 571.3 | 255.2 | negative |
| 18:0 LPI | 599.3 | 283.3 | negative |
| 18:1 LPI | 597.3 | 281.2 | negative |
| 18:2 LPI | 595.3 | 279.2 | negative |
| 17:1 LPI | 583.3 | 267.2 | negative |
| 32:0 PI | 809.5 | 255.2 | negative |
| 34:0 PI | 837.6 | 255.2 | negative |
| 34:1 PI | 835.5 | 255.2 | negative |
| 36:1 PI | 863.6 | 283.3 | negative |
| 18:0-18:2 PI | 861.6 | 283.3 | negative |
| 18:1-18:1 PI | 861.6 | 281.2 | negative |
| 17:0-14:1 PI | 793.5 | 269.2 | negative |

Abbreviations: LPA, lysophosphatidic acid; PA, phosphatidic acid; LPC, lysophosphatidylcholine; PtdCho, phosphatidylcholine; LPS, lysophosphatidylserine; PS, phosphatidylserine; LPG, lysophosphatidylglycerol; PG, phosphatidylglycerol; LPE, lysophosphatidylethanolamine; PE, phosphatidylethanolamine; LPI, lysophosphatidylinositol; PI, phosphatidylinositol.
