# Supplementary material for: EDI3 knockdown in ER-HER2+ breast cancer cells reduces tumor burden and improves survival in two mouse models of experimental metastasis
Source: Breast Cancer Res. 2024 May 30;26:87. doi: 10.1186/s13058-024-01849-y (PMC11138102; doi:10.1186/s13058-024-01849-y)
Supplement: Supplementary file 4 — Additional file 4. Supplementary Figure S1: MALDI-MSI reveals that the mass of 706.54 m/z correlates with HER2 staining. Representative images of HER2 staining (left) and the mass of 706.54 m/z measured using MALDI-MSI (middle) of tumors dissected from pancreas of CD1 nude mice eight weeks after intraperitoneal injection of doxycycline-induced and non-induced HCC1954-luc shEDI3 cells. The mass of 706.54 m/z (candidate molecule PC 30:0) correlated in a large part with HER2 staining and was used to generate tumor-cell-containing areas-of-interest (right). Scale bars represent 1 mm [file 13058_2024_1849_MOESM4_ESM.pdf]

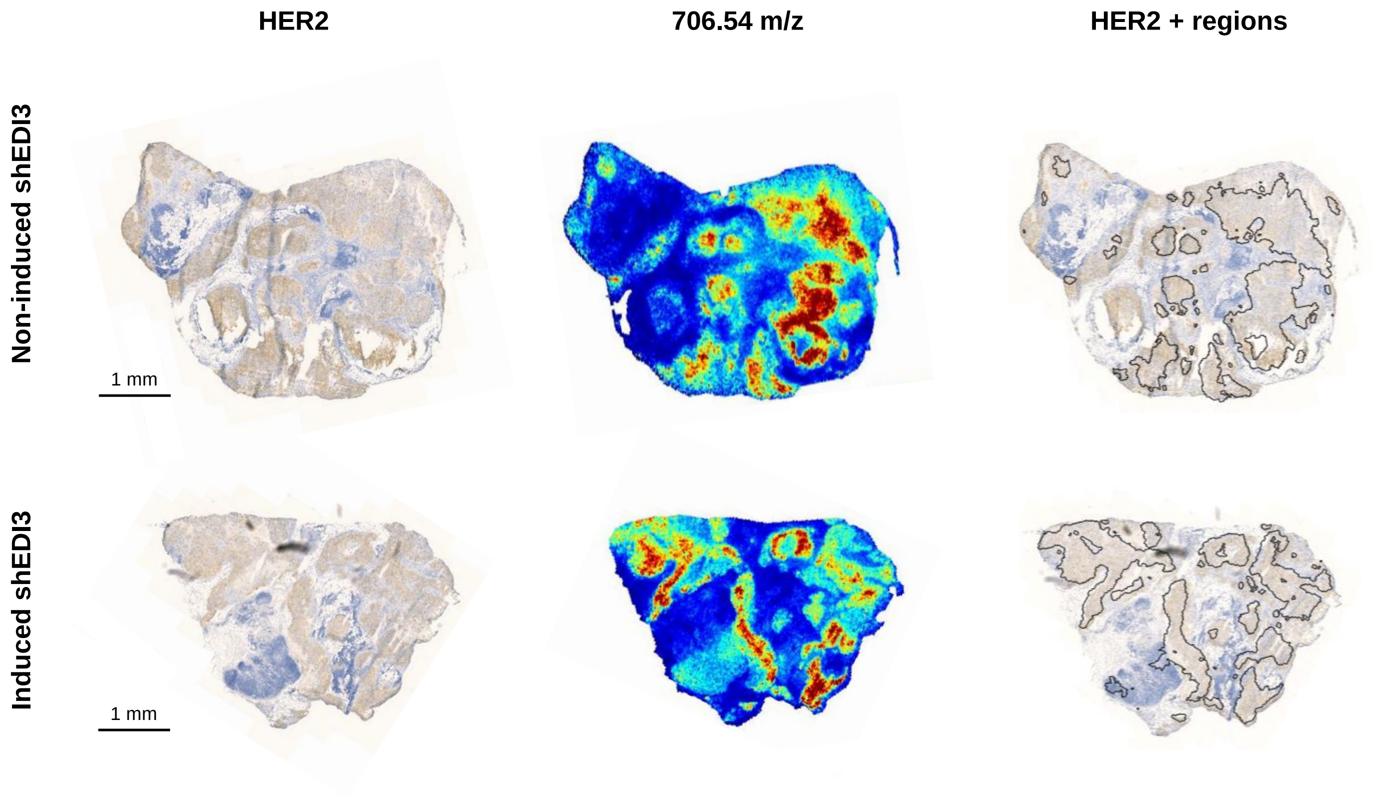

Supplementary Figure S1. MALDI-MSI reveals that the mass of 706.54 m/z correlates with HER2 staining. Representative images of HER2 staining (left) and the mass of 706.54 m/z measured using MALDI-MSI (middle) of tumors dissected from pancreas of CD1 nude mice eight weeks after intraperitoneal injection of doxycycline-induced and non-induced HCC1954-luc shEDI3 cells. The mass of 706.54 m/z (candidate molecule PC 30:0) correlated in a large part with HER2 staining and was used to generate tumor-cell-containing areas-of-interest (right). Scale bars represent 1 mm.
