# Supplementary material for: EDI3 knockdown in ER-HER2+ breast cancer cells reduces tumor burden and improves survival in two mouse models of experimental metastasis
Source: Breast Cancer Res. 2024 May 30;26:87. doi: 10.1186/s13058-024-01849-y (PMC11138102; doi:10.1186/s13058-024-01849-y)
Supplement: Supplementary file 5 — Additional file 5. Supplementary Figure S2: Silencing EDI3 alters intracellular choline metabolite levels in ER-HER2+ breast cancer cells. Intracellular levels of EDI3’s substrate glycerophosphocholine (GPC), as well as EDI3 products choline and G3P (also shown in Figure 1) and choline products phosphocholine (PCho) and betaine measured using LC-MS/MS 72 h after silencing EDI3 (A) inducibly with shRNA in HCC1954 or transiently with siRNA in (B) SUM190PT and (C) SKBR3 breast cancer cells. Metabolite levels were determined by calculating the ratios of the integrated peaks of the endogenous metabolites and the internal standards. Quantities of metabolites were normalized to cell number and presented relative to negative control (shNEG or siNEG). Data represent mean ± SD from at least five technical replicates (wells from a six-well plate) (*p < 0.05; **p < 0.01; ***p < 0.001; ****p < 0.0001) [file 13058_2024_1849_MOESM5_ESM.pptx]

## Slide 1
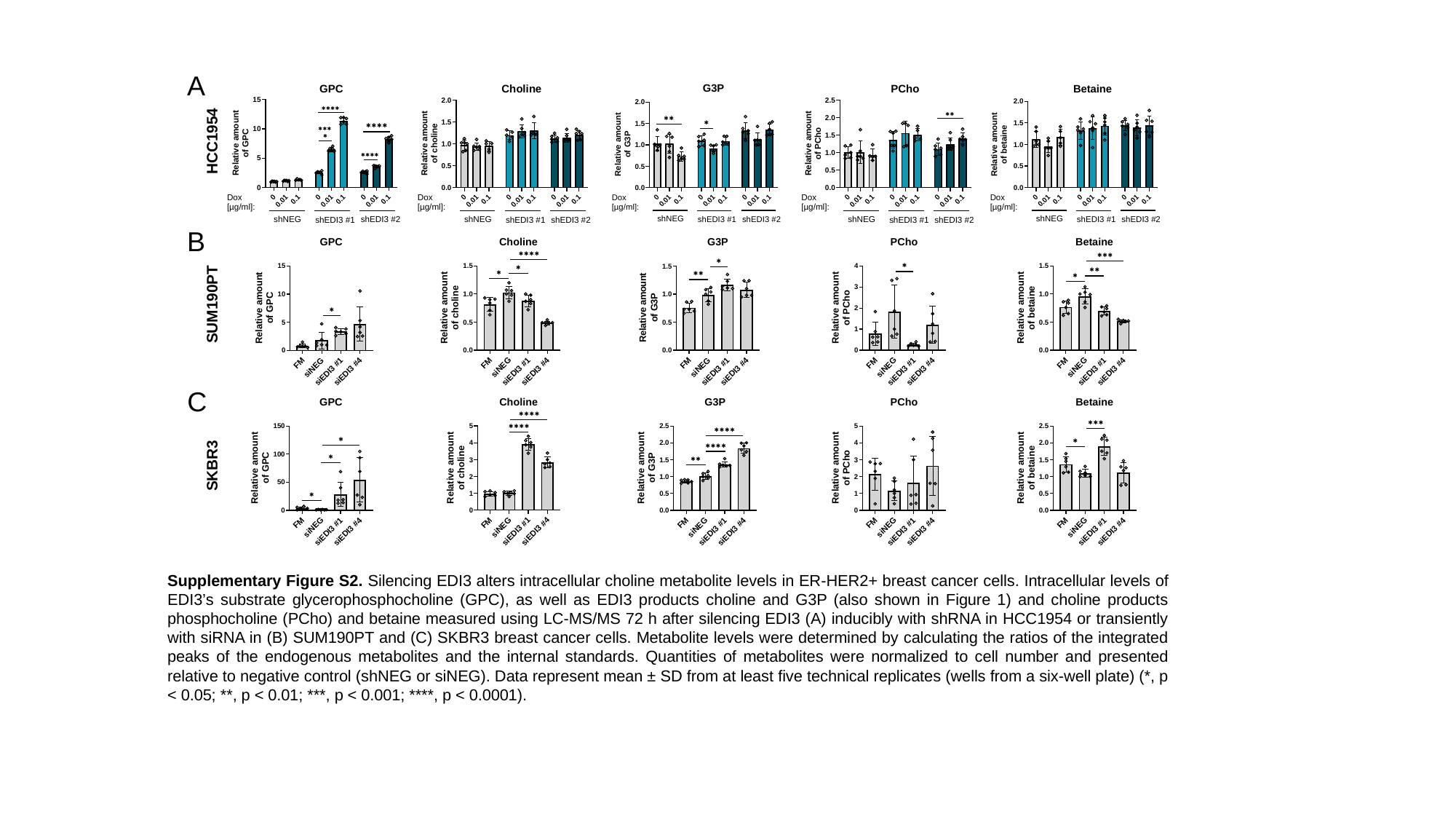

A
HCC1954
B
SUM190PT
C
SKBR3
Supplementary Figure S2. Silencing EDI3 alters intracellular choline metabolite levels in ER-HER2+ breast cancer cells. Intracellular levels of EDI3’s substrate glycerophosphocholine (GPC), as well as EDI3 products choline and G3P (also shown in Figure 1) and choline products phosphocholine (PCho) and betaine measured using LC-MS/MS 72 h after silencing EDI3 (A) inducibly with shRNA in HCC1954 or transiently with siRNA in (B) SUM190PT and (C) SKBR3 breast cancer cells. Metabolite levels were determined by calculating the ratios of the integrated peaks of the endogenous metabolites and the internal standards. Quantities of metabolites were normalized to cell number and presented relative to negative control (shNEG or siNEG). Data represent mean ± SD from at least five technical replicates (wells from a six-well plate) (*, p < 0.05; **, p < 0.01; ***, p < 0.001; ****, p < 0.0001).
