# Supplementary material for: EDI3 knockdown in ER-HER2+ breast cancer cells reduces tumor burden and improves survival in two mouse models of experimental metastasis
Source: Breast Cancer Res. 2024 May 30;26:87. doi: 10.1186/s13058-024-01849-y (PMC11138102; doi:10.1186/s13058-024-01849-y)
Supplement: Supplementary file 6 — Additional file 6. Supplementary Figure S3: EDI3 knockdown in SUM190PT and SKBR3 cells used for metabolite analysis. EDI3 mRNA expression and Western blots with corresponding quantification showing EDI3 protein expression after silencing EDI3 in (A) SUM190PT and (B) SKBR3 breast cancer cells compared with cells transfected with scrambled siRNA (siNEG #1). Replicates of these cells were used for the analysis of intracellular choline metabolites and lipids by LC-MS/MS. FM, full media control [file 13058_2024_1849_MOESM6_ESM.pptx]

## Slide 1
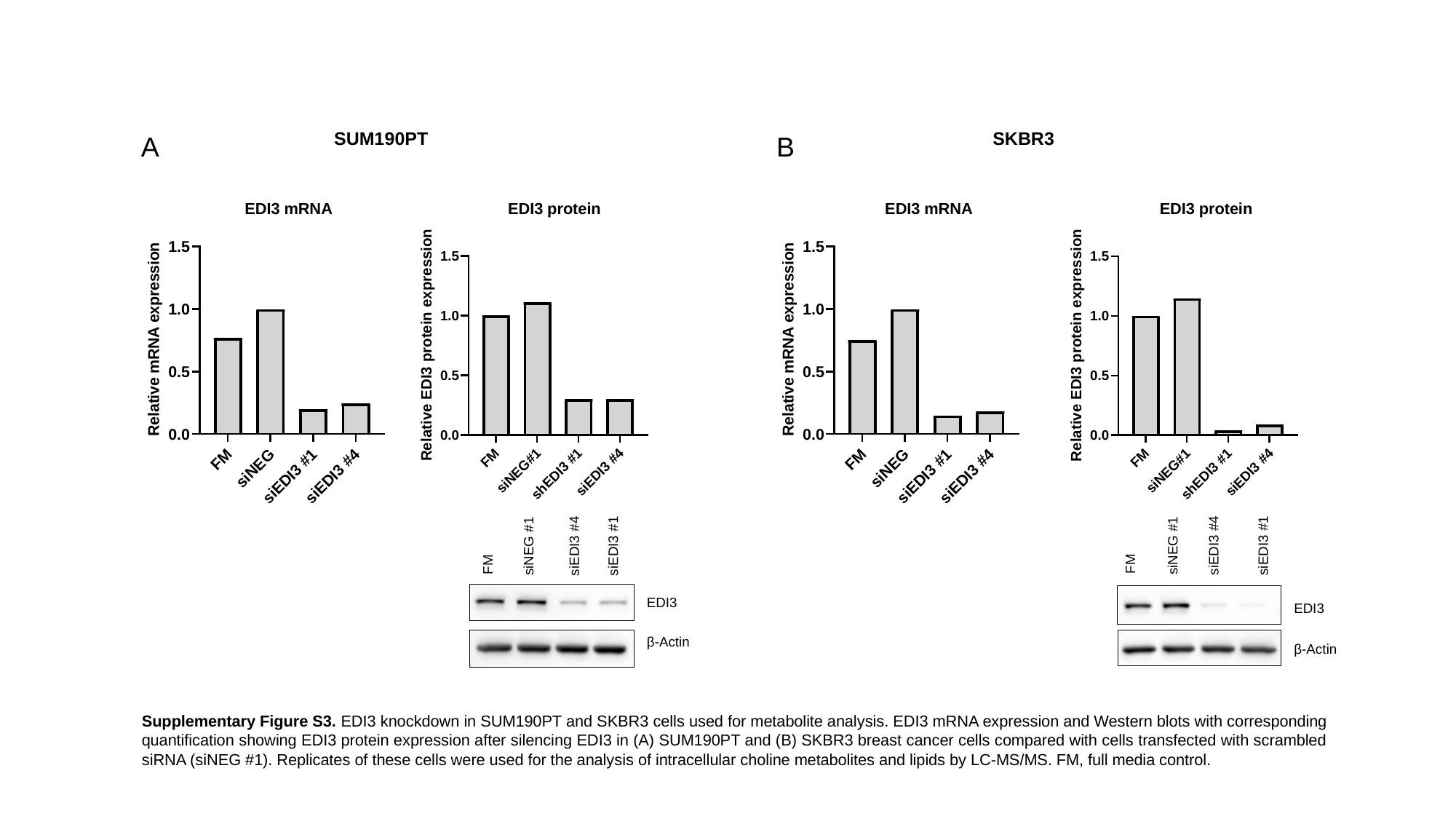

SUM190PT
SKBR3
A
B
EDI3 mRNA
EDI3 protein
EDI3 mRNA
EDI3 protein
siEDI3 #4
siEDI3 #1
siNEG #1
siEDI3 #4
siEDI3 #1
siNEG #1
FM
FM
EDI3
EDI3
β-Actin
β-Actin
Supplementary Figure S3. EDI3 knockdown in SUM190PT and SKBR3 cells used for metabolite analysis. EDI3 mRNA expression and Western blots with corresponding quantification showing EDI3 protein expression after silencing EDI3 in (A) SUM190PT and (B) SKBR3 breast cancer cells compared with cells transfected with scrambled siRNA (siNEG #1). Replicates of these cells were used for the analysis of intracellular choline metabolites and lipids by LC-MS/MS. FM, full media control.
