# Supplementary material for: EDI3 knockdown in ER-HER2+ breast cancer cells reduces tumor burden and improves survival in two mouse models of experimental metastasis
Source: Breast Cancer Res. 2024 May 30;26:87. doi: 10.1186/s13058-024-01849-y (PMC11138102; doi:10.1186/s13058-024-01849-y)
Supplement: Supplementary file 7 — Additional file 7. Supplementary Figure S4: Silencing EDI3 alters glycerophospholipid levels in ER-HER2+ breast cancer cells. Intracellular levels of different species of LPA, PA, LPC, PtdCho, LPS, PS, LPG, PG, LPE, PE, LPI and PI were measured using LC-MS/MS 72 h after silencing EDI3 either inducibly with shRNA in HCC1954 or transiently with siRNA in SUM190PT and SKBR3 breast cancer cells. Metabolite levels were determined by calculating the ratios of the integrated peaks of the endogenous metabolites and the internal standards. Quantities of metabolites were normalized to cell number and presented relative to negative control (shNEG or siNEG). Data represent mean ± SD from at least five technical replicates (wells from a six-well plate) from one or two independent experiments (*p < 0.05; **p < 0.01; ***p < 0.001; ****p < 0.0001). LPA, lysophosphatidic acid; PA, phosphatidic acid; LPC, lysophosphatidylcholine; PtdCho, phosphatidylcholine; LPS, lysophosphatidylserine; PS, phosphatidylserine; LPG, lysophosphatidylglycerol; PG, phosphatidylglycerol; LPE, lysophosphatidylethanolamine; PE, phosphatidylethanolamine; LPI, lysophosphatidylinositol; PI, phosphatidylinositol [file 13058_2024_1849_MOESM7_ESM.pdf]

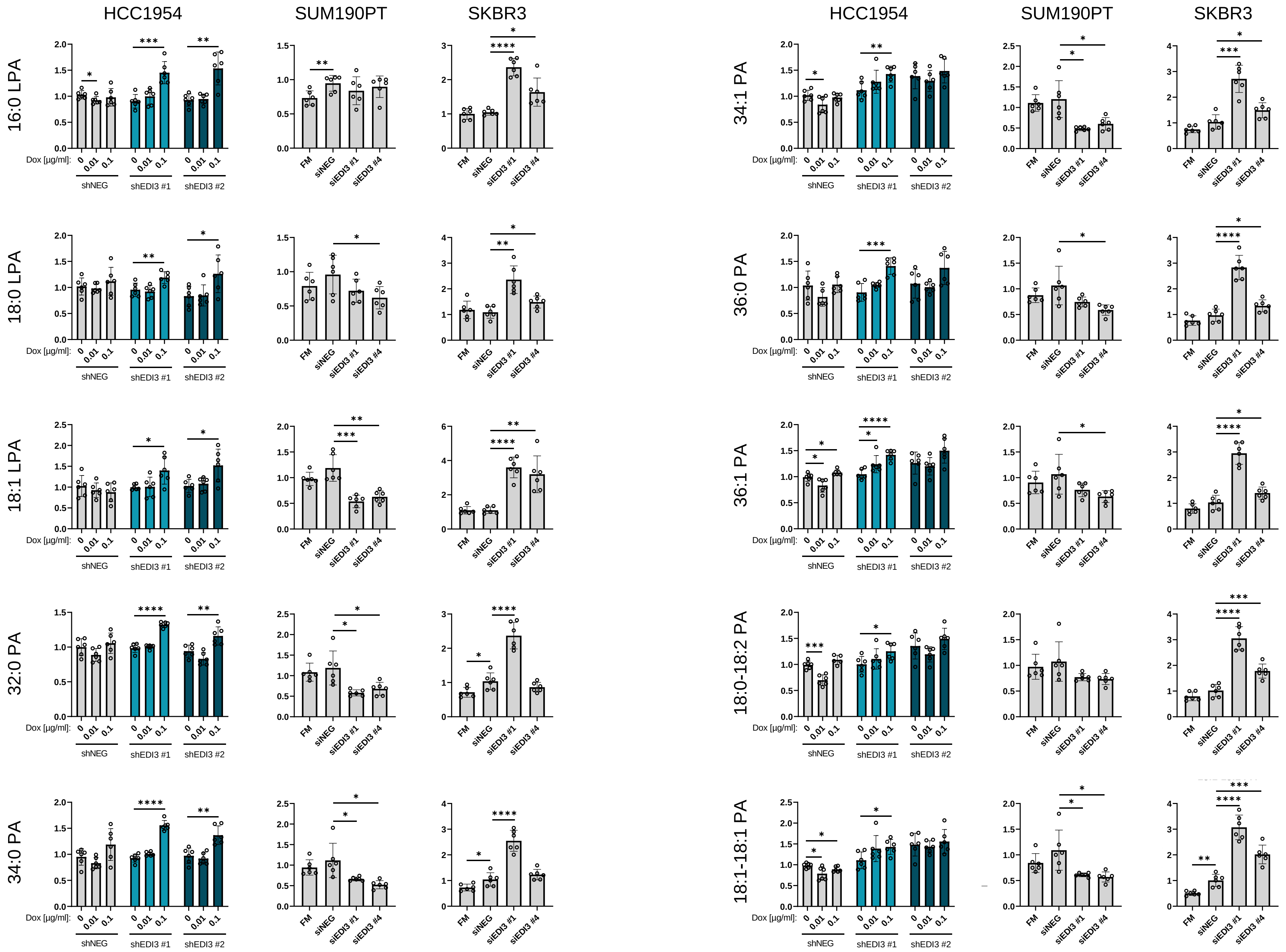

HCC1954
SUM190PT
SKBR3
HCC1954
SUM190PT
SKBR3
16:0 LPA
18:0 LPA
18:1 LPA
32:0 PA
34:0 PA
34:1 PA
36:0 PA
36:1 PA
18:0-18:2 PA
18:1-18:1 PA
Dox [µg/ml]:
0
0.01
0.1
shNEG
shEDI3 #1
shEDI3 #2
FM
siNEG
siEDI3 #1
siEDI3 #4

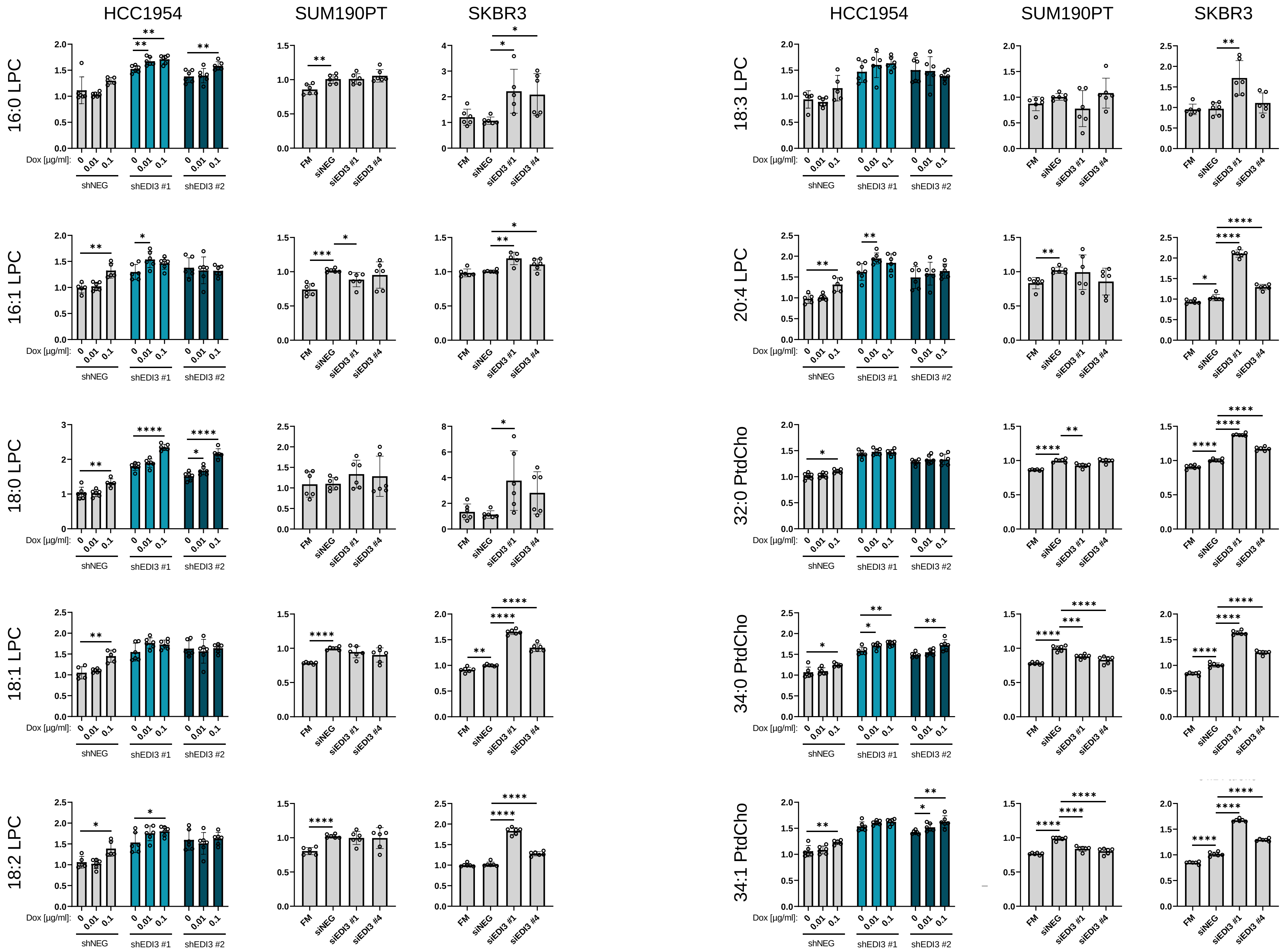

HCC1954
SUM190PT
SKBR3
HCC1954
SUM190PT
SKBR3
16:0 LPC
16:1 LPC
18:0 LPC
18:1 LPC
18:2 LPC
18:3 LPC
20:4 LPC
32:0 PtdCho
34:0 PtdCho
34:1 PtdCho
Dox [µg/ml]:
0
0.01
0.1
shNEG
shEDI3 #1
shEDI3 #2
FM
siNEG
siEDI3 #1
siEDI3 #4

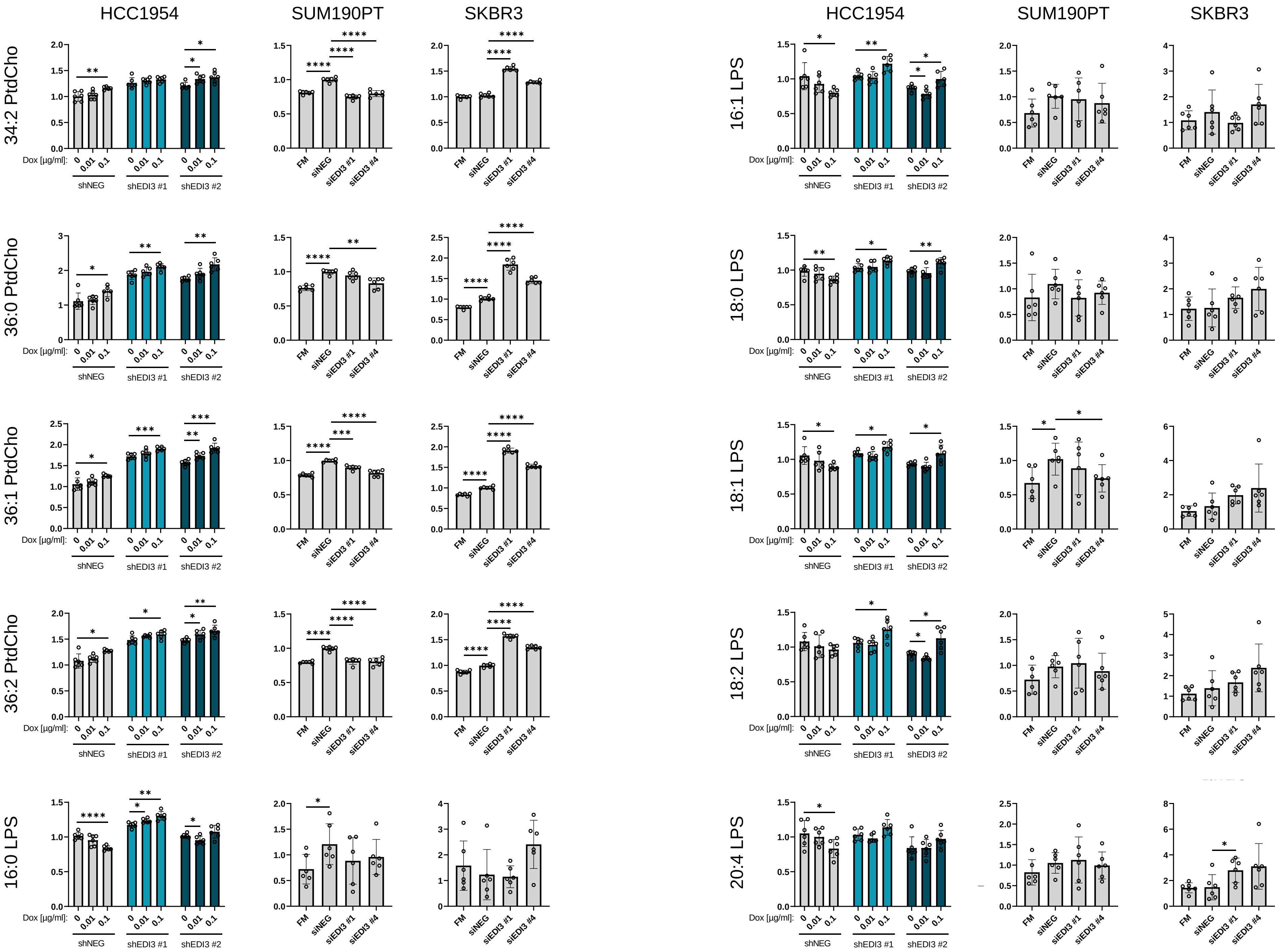

HCC1954
SUM190PT
SKBR3
HCC1954
SUM190PT
SKBR3
34:2 PtdCho
36:0 PtdCho
36:1 PtdCho
36:2 PtdCho
16:0 LPS
16:1 LPS
18:0 LPS
18:1 LPS
18:2 LPS
20:4 LPS
Dox [µg/ml]:
0
0.01
0.1
shNEG
shEDI3 #1
shEDI3 #2
FM
siNEG
siEDI3 #1
siEDI3 #4

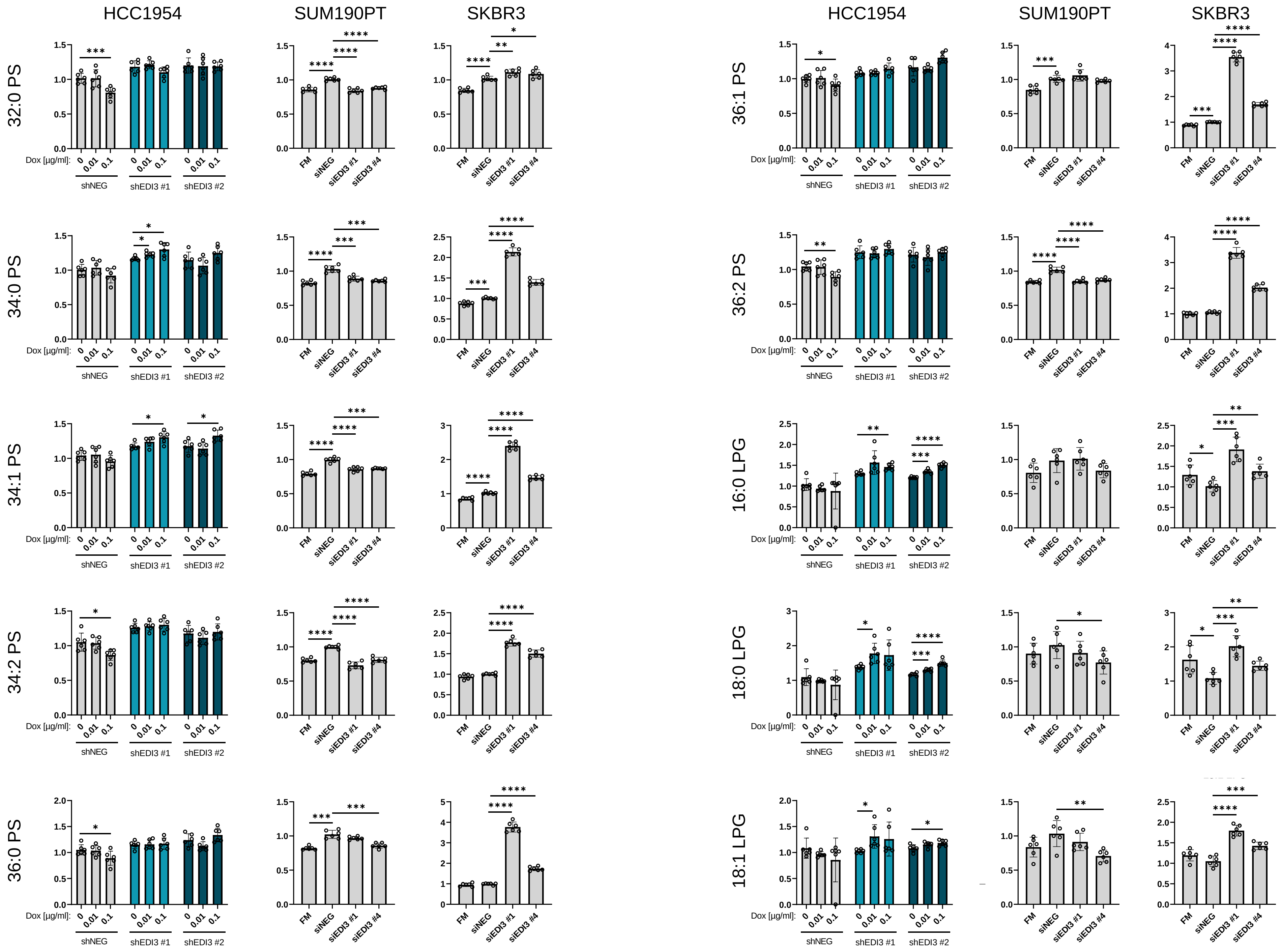

HCC1954
SUM190PT
SKBR3
HCC1954
SUM190PT
SKBR3
32:0 PS
34:0 PS
34:1 PS
34:2 PS
36:0 PS
36:1 PS
36:2 PS
16:0 LPG
18:0 LPG
18:1 LPG
Dox [µg/ml]:
0
0.01
0.1
shNEG
shEDI3 #1
shEDI3 #2
FM
siNEG
siEDI3 #1
siEDI3 #4

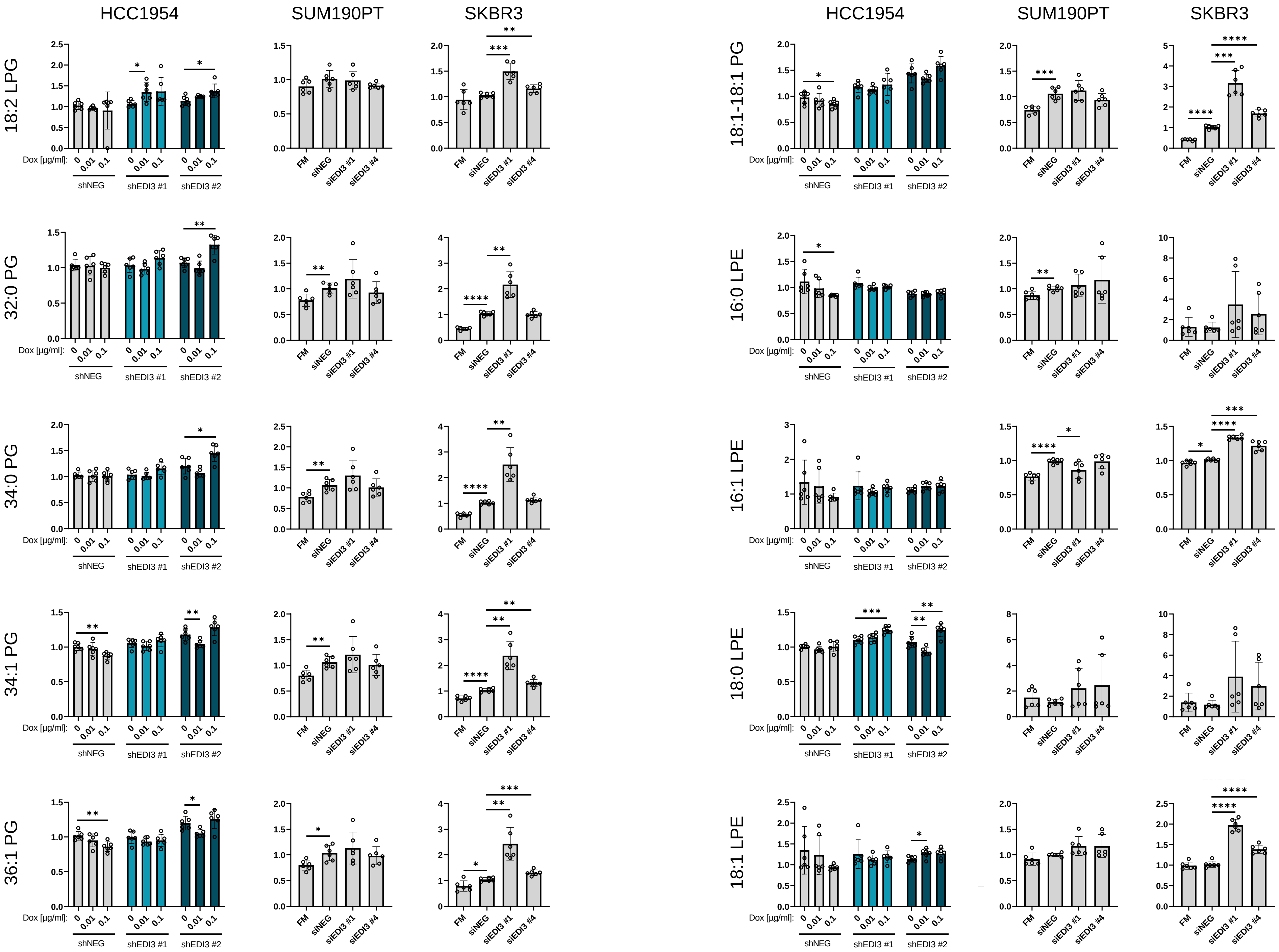

HCC1954
SUM190PT
SKBR3
HCC1954
SUM190PT
SKBR3
18:2 LPG
32:0 PG
34:0 PG
34:1 PG
36:1 PG
18:1-18:1 PG
16:0 LPE
16:1 LPE
18:0 LPE
18:1 LPE
Dox [µg/ml]:
0
0.01
0.1
shNEG
shEDI3 #1
shEDI3 #2
FM
siNEG
siEDI3 #1
siEDI3 #4

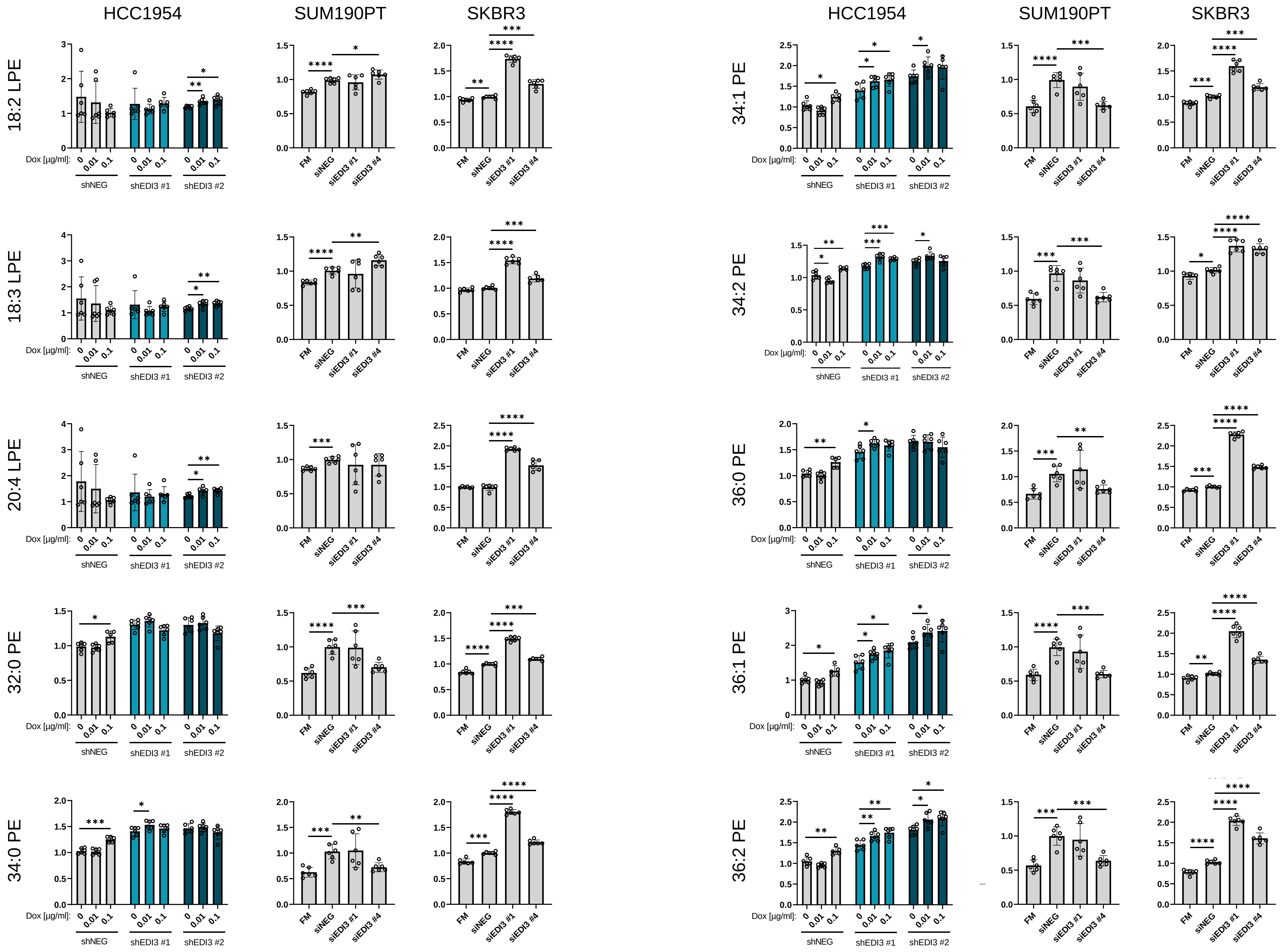

HCC1954
SUM190PT
SKBR3
18:2 LPE
18:3 LPE
20:4 LPE
32:0 PE
34:0 PE
34:1 PE
34:2 PE
36:0 PE
36:1 PE
36:2 PE
Dox [µg/ml]:
0
0.01
0.1
shNEG
shEDI3 #1
shEDI3 #2
FM
siNEG
siEDI3 #1
siEDI3 #4

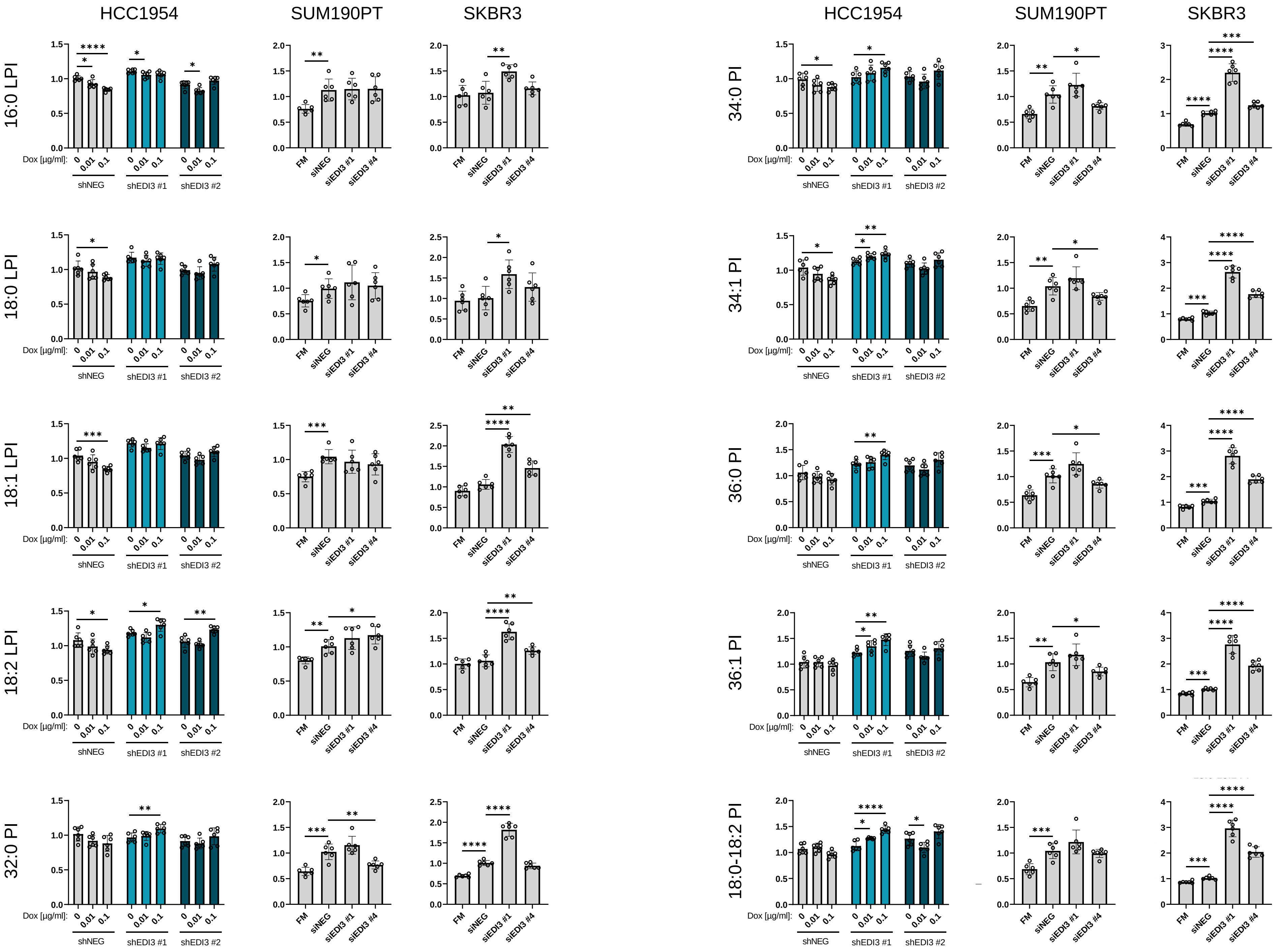
HCC1954
SUM190PT
SKBR3
16:0 LPI
18:0 LPI
18:1 LPI
18:2 LPI
32:0 PI
34:0 PI
34:1 PI
36:0 PI
36:1 PI
18:0-18:2 PI
Dox [µg/ml]:
0
0.01
0.1
shNEG
shEDI3 #1
shEDI3 #2
FM
siNEG
siEDI3 #1
siEDI3 #4

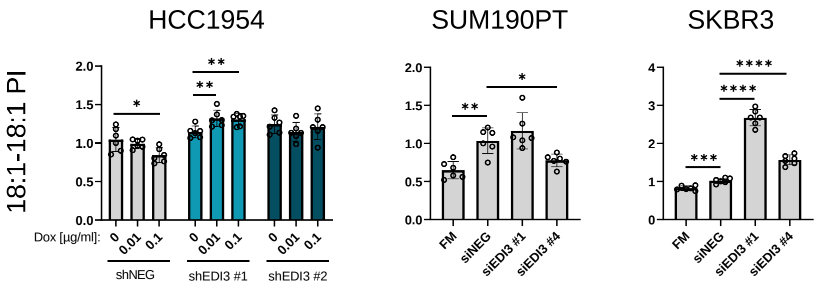

Supplementary Figure S4 Silencing EDI3 alters glycerophospholipid levels in ER-HER2+ breast cancer cells. Intracellular levels of different species of LPA, PA, LPC, PtdCho, LPS, PS, LPG, PG, LPE, PE, LPI and PI were measured using LC-MS/MS 72 h after silencing EDI3 either inducibly with shRNA in HCC1954 or transiently with siRNA in SUM190PT and SKBR3 breast cancer cells. Metabolite levels were determined by calculating the ratios of the integrated peaks of the endogenous metabolites and the internal standards. Quantities of metabolites were normalized to cell number and presented relative to negative control (shNEG or siNEG). Data represent mean ± SD from at least five technical replicates (wells from a six-well plate) from one or two independent experiments (*, $p < 0.05$; **, $p < 0.01$; ***, $p < 0.001$; ****, $p < 0.0001$). LPA, lysophosphatidic acid; PA, phosphatidic acid; LPC, lysophosphatidylcholine; PtdCho, phosphatidylcholine; LPS, lysophosphatidylserine; PS, phosphatidylserine; LPG, lysophosphatidylglycerol; PG, phosphatidylglycerol; LPE, lysophosphatidylethanolamine; PE, phosphatidylethanolamine; LPI, lysophosphatidylinositol; PI, phosphatidylinositol.
