# Supplementary material for: EDI3 knockdown in ER-HER2+ breast cancer cells reduces tumor burden and improves survival in two mouse models of experimental metastasis
Source: Breast Cancer Res. 2024 May 30;26:87. doi: 10.1186/s13058-024-01849-y (PMC11138102; doi:10.1186/s13058-024-01849-y)
Supplement: Supplementary file 8 — Additional file 8. Supplementary Figure S5: EDI3 silencing using siRNA does not influence migration in ER-HER2+ HCC1954 cells. Percentage of wound closure relative to negative control (siNEG) after silencing EDI3 with three siRNA oligos targeting different exons in HCC1954 cells. Values in graph represent mean ± SD from two independent experiments. FM, full media control [file 13058_2024_1849_MOESM8_ESM.pptx]

## Slide 1
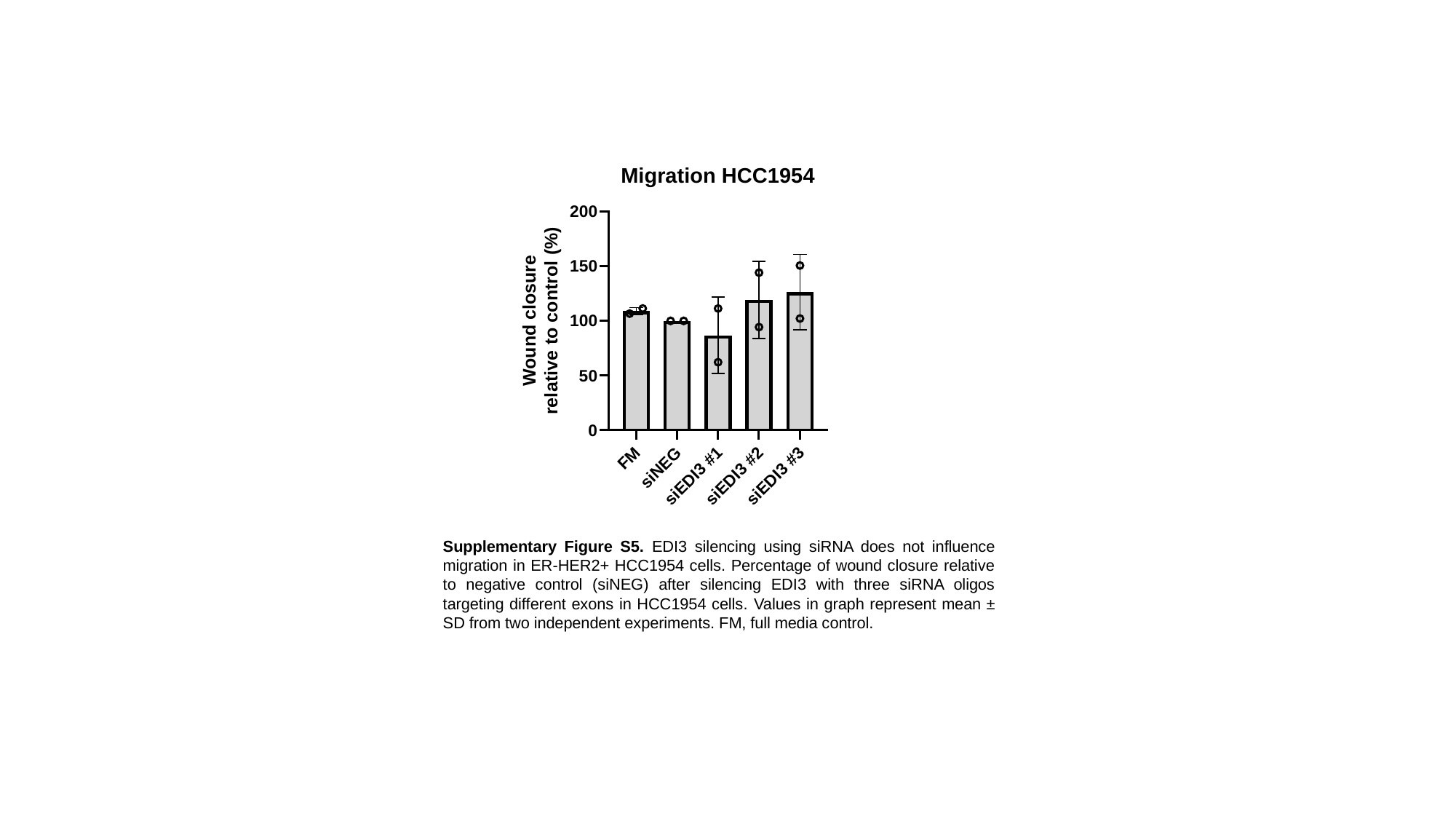

Supplementary Figure S5. EDI3 silencing using siRNA does not influence migration in ER-HER2+ HCC1954 cells. Percentage of wound closure relative to negative control (siNEG) after silencing EDI3 with three siRNA oligos targeting different exons in HCC1954 cells. Values in graph represent mean ± SD from two independent experiments. FM, full media control.
