# Supplementary material for: EDI3 knockdown in ER-HER2+ breast cancer cells reduces tumor burden and improves survival in two mouse models of experimental metastasis
Source: Breast Cancer Res. 2024 May 30;26:87. doi: 10.1186/s13058-024-01849-y (PMC11138102; doi:10.1186/s13058-024-01849-y)
Supplement: Supplementary file 9 — Additional file 9. Supplementary Figure S6: Replenishing cell media with fresh doxycycline does not additionally affect EDI3 expression nor colony formation. Non-induced HCC1954_Luc shEDI3 cells were plated for colony formation assay in media containing 0 µg/ml or 0.1 µg/ml doxycycline. Media was either not changed (-mc) over the assay period of 14 days or media was replenished +/- fresh doxycycline every 3 days (+mc). A) EDI3 mRNA expression after 14 days of colony formation assay both with and without media change. B) Representative images (left) and corresponding quantification of colony number (middle) and size (right). Values in graphs represent mean ± SD from three independent experiments (*p < 0.05; **p < 0.01; ns, not significant) [file 13058_2024_1849_MOESM9_ESM.pptx]

## Slide 1
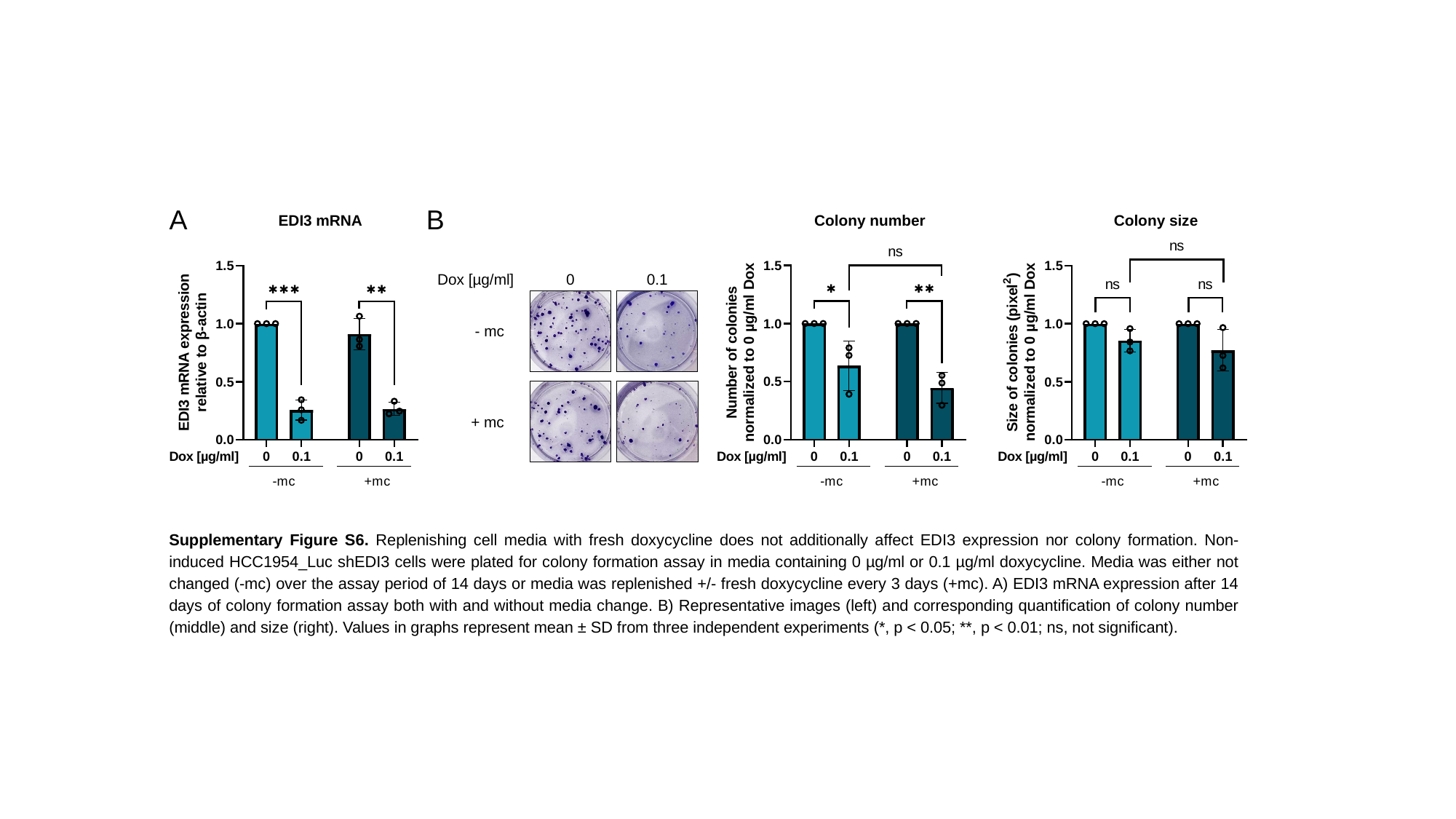

A
B
EDI3 mRNA
Colony number
Colony size
Dox [µg/ml]
0
0.1
- mc
+ mc
Supplementary Figure S6. Replenishing cell media with fresh doxycycline does not additionally affect EDI3 expression nor colony formation. Non-induced HCC1954_Luc shEDI3 cells were plated for colony formation assay in media containing 0 µg/ml or 0.1 µg/ml doxycycline. Media was either not changed (-mc) over the assay period of 14 days or media was replenished +/- fresh doxycycline every 3 days (+mc). A) EDI3 mRNA expression after 14 days of colony formation assay both with and without media change. B) Representative images (left) and corresponding quantification of colony number (middle) and size (right). Values in graphs represent mean ± SD from three independent experiments (*, p < 0.05; **, p < 0.01; ns, not significant).
