# Supplementary material for: EDI3 knockdown in ER-HER2+ breast cancer cells reduces tumor burden and improves survival in two mouse models of experimental metastasis
Source: Breast Cancer Res. 2024 May 30;26:87. doi: 10.1186/s13058-024-01849-y (PMC11138102; doi:10.1186/s13058-024-01849-y)
Supplement: Supplementary file 10 — Additional file 10. Supplementary Figure S7: Silencing EDI3 in SUM190PT cells using siRNA reduces resistance to anoikis and viability in adherent cells. (A) EDI3 mRNA and (B) protein expression after silencing EDI3 in SUM190PT cells compared with cells transfected with two different scrambled siRNA (siNEG #1 and #2). Viability in RFU relative to negative control measured in (C) non-adherent cells 48 h after plating on a poly-HEMA matrix or in (D) adherent cells 96 h after plating. Data represent mean ± SD from three independent experiments (*p < 0.05; ***p < 0.001). FM, full media control; RFU, relative fluorescence units [file 13058_2024_1849_MOESM10_ESM.pptx]

## Slide 1
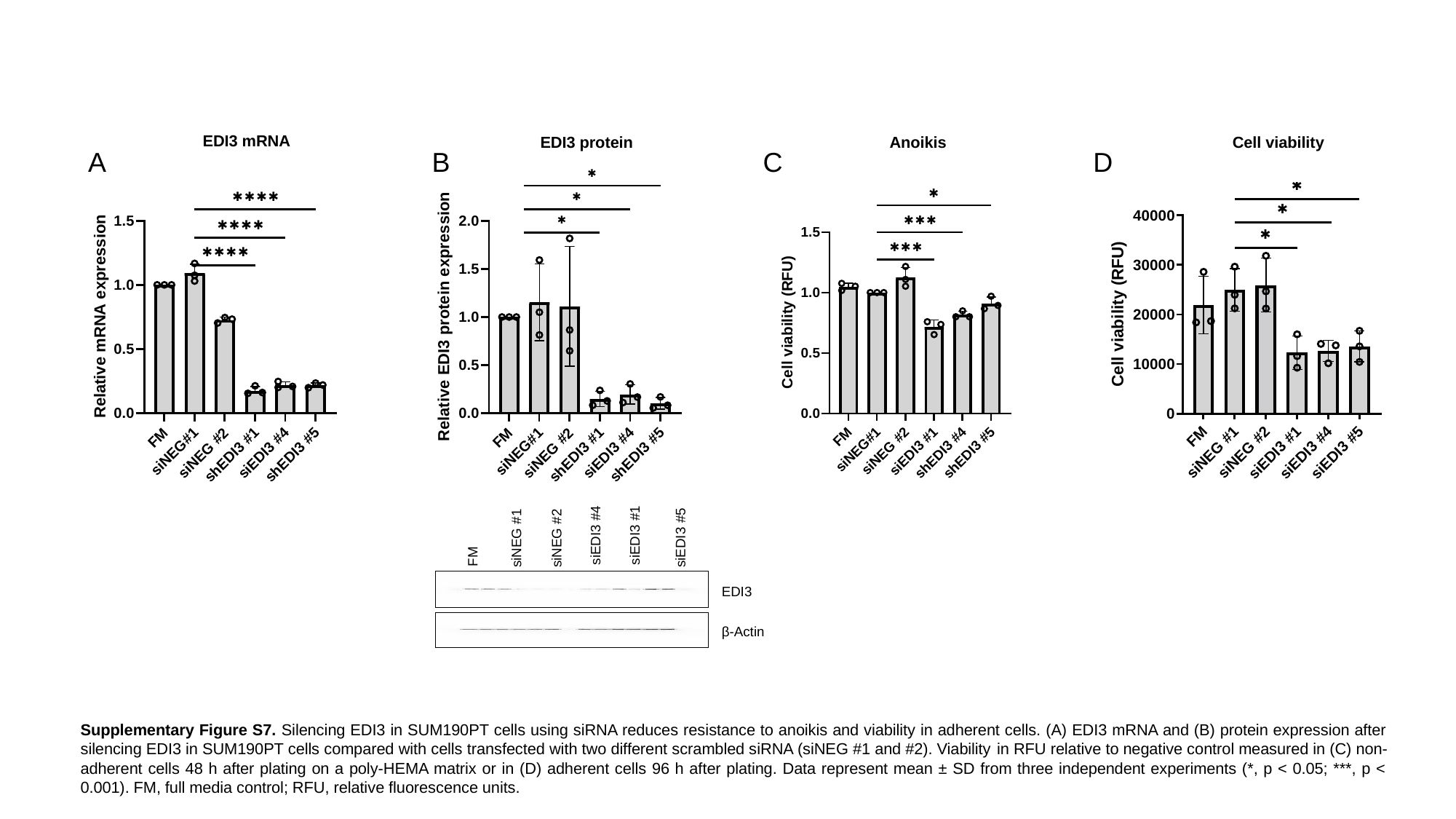

EDI3 mRNA
EDI3 protein
Anoikis
Cell viability
A
B
C
D
siEDI3 #4
siEDI3 #1
siEDI3 #5
siNEG #1
siNEG #2
FM
EDI3
β-Actin
Supplementary Figure S7. Silencing EDI3 in SUM190PT cells using siRNA reduces resistance to anoikis and viability in adherent cells. (A) EDI3 mRNA and (B) protein expression after silencing EDI3 in SUM190PT cells compared with cells transfected with two different scrambled siRNA (siNEG #1 and #2). Viability in RFU relative to negative control measured in (C) non-adherent cells 48 h after plating on a poly-HEMA matrix or in (D) adherent cells 96 h after plating. Data represent mean ± SD from three independent experiments (*, p < 0.05; ***, p < 0.001). FM, full media control; RFU, relative fluorescence units.
