# Supplementary material for: EDI3 knockdown in ER-HER2+ breast cancer cells reduces tumor burden and improves survival in two mouse models of experimental metastasis
Source: Breast Cancer Res. 2024 May 30;26:87. doi: 10.1186/s13058-024-01849-y (PMC11138102; doi:10.1186/s13058-024-01849-y)
Supplement: Supplementary file 11 — Additional file 11: Supplementary Figure S8: Luminescence signals detected in lungs after tail vein injection of HCC1954-luc cells decrease over time. (A) Luciferase-expressing HCC1954 shNEG and shEDI3 cells were treated with doxycycline for 72 h, followed by tail vein injection into doxycycline pre-treated and untreated CD1 nude mice, respectively. In contrast to the images of the same mice presented in Figure 3, where the luminescent signals from induced and non-induced mice were compared to one another each week, here, luminescence signals are shown on one scale to visualize how the signal intensity declines over the period of six weeks from time 0. Doxycycline was administered to the mice by a 625 mg/kg doxycycline containing diet (Ssniff) ad libitum. Representative luminescence images of five mice per condition are shown. (B) Luminescence signal remains stable in HCC1954-luc cells for 15 weeks, even in the absence of antibiotic G418 which is needed for selection and maintenance of positively transduced clones. This was an important control for the in vivo experiments, as the cells were no longer under the selection pressure of G418 once injected into mice. Luciferase assay was performed over time with HCC1954-luc cells cultured +/- G418 for up to 15 weeks. Measurements represent mean ± SD of three technical replicates. (C) Luminescence signal 13 weeks after intraperitoneal (IP) injection of HCC1954-luc shEDI3 in CD1 nude mice confirms the presence of tumor cells in mice that were used to assess survival time. Luminescence was measured 3 min after administration of luciferin [file 13058_2024_1849_MOESM11_ESM.pptx]

## Slide 1
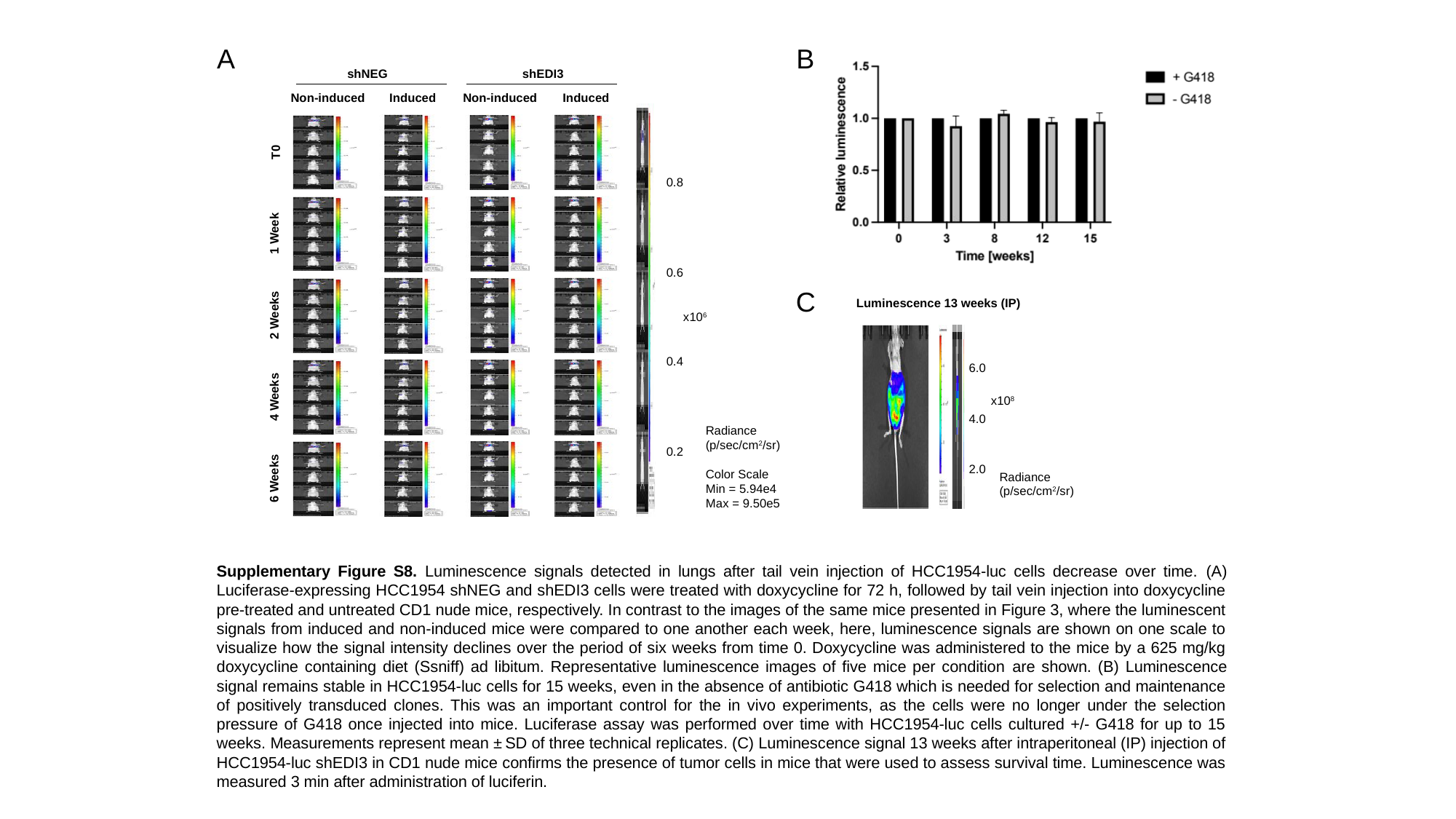

A
B
shNEG
shEDI3
Non-induced
Induced
Non-induced
Induced
T0
0.8
1 Week
0.6
2 Weeks
x106
0.4
4 Weeks
Radiance
(p/sec/cm2/sr)
0.2
Color Scale
Min = 5.94e4
Max = 9.50e5
6 Weeks
C
Luminescence 13 weeks (IP)
6.0
x108
4.0
2.0
Radiance
(p/sec/cm2/sr)
Supplementary Figure S8. Luminescence signals detected in lungs after tail vein injection of HCC1954-luc cells decrease over time. (A) Luciferase-expressing HCC1954 shNEG and shEDI3 cells were treated with doxycycline for 72 h, followed by tail vein injection into doxycycline pre-treated and untreated CD1 nude mice, respectively. In contrast to the images of the same mice presented in Figure 3, where the luminescent signals from induced and non-induced mice were compared to one another each week, here, luminescence signals are shown on one scale to visualize how the signal intensity declines over the period of six weeks from time 0. Doxycycline was administered to the mice by a 625 mg/kg doxycycline containing diet (Ssniff) ad libitum. Representative luminescence images of five mice per condition are shown. (B) Luminescence signal remains stable in HCC1954-luc cells for 15 weeks, even in the absence of antibiotic G418 which is needed for selection and maintenance of positively transduced clones. This was an important control for the in vivo experiments, as the cells were no longer under the selection pressure of G418 once injected into mice. Luciferase assay was performed over time with HCC1954-luc cells cultured +/- G418 for up to 15 weeks. Measurements represent mean ± SD of three technical replicates. (C) Luminescence signal 13 weeks after intraperitoneal (IP) injection of HCC1954-luc shEDI3 in CD1 nude mice confirms the presence of tumor cells in mice that were used to assess survival time. Luminescence was measured 3 min after administration of luciferin.
