# Supplementary material for: EDI3 knockdown in ER-HER2+ breast cancer cells reduces tumor burden and improves survival in two mouse models of experimental metastasis
Source: Breast Cancer Res. 2024 May 30;26:87. doi: 10.1186/s13058-024-01849-y (PMC11138102; doi:10.1186/s13058-024-01849-y)
Supplement: Supplementary file 12 — Additional file 12. Supplementary Figure S9: In vivo bioluminescence imaging confirms reduced peritoneal metastases in mice prior to organ collection. HCC1954-luc shEDI3 cells were induced with doxycycline for 72 h. Induced and non-induced cells were injected into the peritoneum of doxycycline pre-treated and untreated CD1 nude mice, respectively. Doxycycline was administered to the mice by a diet containing 625 mg/kg doxycycline (Ssniff) ad libitum. (A) EDI3 mRNA expression and (B) corresponding Western blot showing EDI3 protein expression in the cells at time of injection. (C) In vivo luminescence images acquired six and eight weeks after injection (left) and corresponding quantitative analysis of luminescence signal normalized to T0 (right). Data in (C) represent seven mice per condition. Box plots: horizontal line, median; box, 25th-75th percentiles; whiskers, min to max (*p < 0.05) [file 13058_2024_1849_MOESM12_ESM.pptx]

## Slide 1
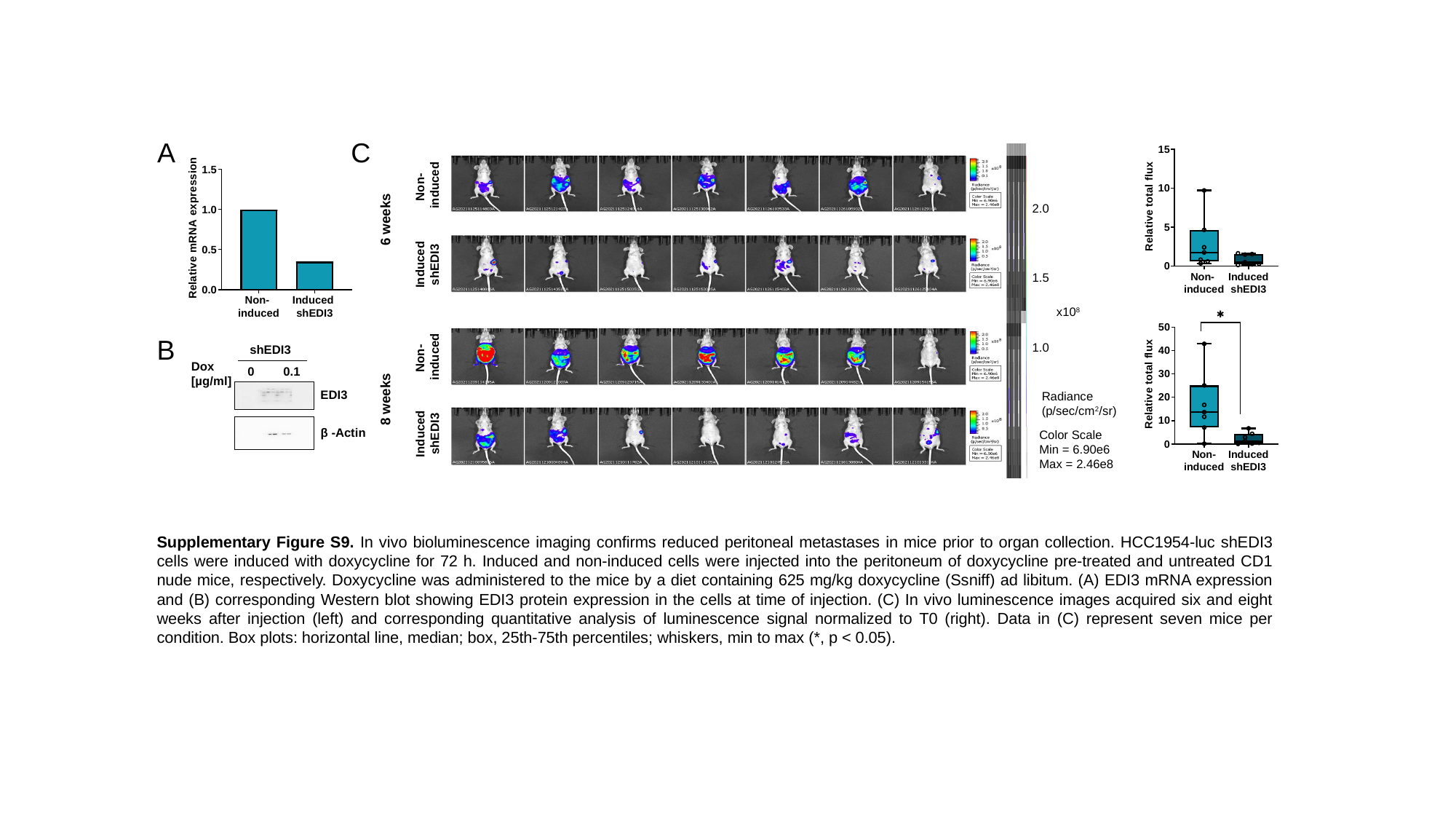

A
C
Non-
induced
2.0
6 weeks
Induced shEDI3
1.5
x108
B
1.0
Non-
induced
shEDI3
Dox
[µg/ml]
0
0.1
EDI3
Radiance
(p/sec/cm2/sr)
8 weeks
Induced shEDI3
β -Actin
Color Scale
Min = 6.90e6
Max = 2.46e8
Supplementary Figure S9. In vivo bioluminescence imaging confirms reduced peritoneal metastases in mice prior to organ collection. HCC1954-luc shEDI3 cells were induced with doxycycline for 72 h. Induced and non-induced cells were injected into the peritoneum of doxycycline pre-treated and untreated CD1 nude mice, respectively. Doxycycline was administered to the mice by a diet containing 625 mg/kg doxycycline (Ssniff) ad libitum. (A) EDI3 mRNA expression and (B) corresponding Western blot showing EDI3 protein expression in the cells at time of injection. (C) In vivo luminescence images acquired six and eight weeks after injection (left) and corresponding quantitative analysis of luminescence signal normalized to T0 (right). Data in (C) represent seven mice per condition. Box plots: horizontal line, median; box, 25th-75th percentiles; whiskers, min to max (*, p < 0.05).
